# Natural expression variation for the Arabidopsis *MED20a* mediator complex subunit influences quantitative resistance to *Sclerotinia sclerotiorum*

**DOI:** 10.3389/fpls.2025.1706963

**Published:** 2025-11-17

**Authors:** William Underwood, Roshan Sharma Poudel

**Affiliations:** 1 Edward T. Schafer Agricultural Research Center, Sunflower Improvement Research Unit, USDA Agricultural Research Service, Fargo, ND, United States; 2 Department of Plant Pathology, North Dakota State University, Fargo, ND, United States

**Keywords:** white mold, transcriptional mediator, quantitative disease resistance, genome-wide association, resistance mechanisms

## Abstract

**Introduction:**

The necrotrophic fungus *Sclerotinia sclerotiorum* is a destructive plant pathogen that can infect a broad range of host plants, including many agriculturally important crop species. Resistance to *S. sclerotiorum* is partial and quantitative, controlled by many genes. The identities of genes influencing resistance and the molecular mechanisms governing defense against this pathogen are poorly understood. To improve understanding of resistance, we performed genome-wide association studies of *Arabidopsis thaliana* response to inoculation with two isolates of the pathogen differing in aggressiveness and sought to validate our results by identifying the causal gene at a single mapped locus.

**Methods:**

A total of 325 *A. thaliana* ecotypes were evaluated for resistance at two timepoints after inoculation with *S. sclerotiorum* isolate 1980 or BN325. Genome-wide association studies were carried out using two different models to identify loci associated with resistance. *A. thaliana* mutant lines were then evaluated for candidate genes at a single locus to identify the most likely candidate gene influencing resistance, and sequencing of the candidate gene and promoter region was performed to identify putative causal variants.

**Results and discussion:**

Genome-wide association studies mapped 30 loci associated with resistance to *S. sclerotiorum*. Surprisingly, correlations for response to the two isolates among *A. thaliana* ecotypes were relatively weak and no overlapping loci were mapped for resistance to both isolates. *A. thaliana med20a* mutants impaired in a subunit of the transcriptional Mediator complex were more susceptible to *S. sclerotiorum* and a single variant upstream of the *MED20a* gene was associated with resistance. These results improve our mechanistic understanding of resistance to this important plant pathogen.

## Introduction

The necrotrophic fungal pathogen *Sclerotinia sclerotiorum* causes disease on a broad range of primarily dicot plant species, significantly impacting global agricultural production. This fungus infects numerous crop plants such as soybeans, canola, sunflowers, lettuce, and dry edible beans. *S. sclerotiorum* can also infect model plant species such as *Arabidopsis thaliana* and *Nicotiana benthamiana*, allowing for the use of research tools developed for these model species to study genes and molecular mechanisms underlying resistance to this pathogen. Complete resistance to *S. sclerotiorum* has not been identified in host plant species. Instead, resistance is partial and quantitative, conditioned by many genes exerting relatively small effects on the overall level of partial resistance (Bolton et al., 2006; Wang et al., 2019; Derbyshire et al., 2022). The quantitative nature of resistance to this pathogen has made breeding for crop varieties with high levels of resistance challenging and has hindered efforts to identify genes underlying resistance.

As noted above, the model plant species *A. thaliana* is susceptible to *S. sclerotiorum* and has been used extensively to study resistance to this necrotrophic pathogen. The use of *A. thaliana* as a model to elucidate mechanisms of resistance to *S. sclerotiorum* initially focused on studies of known mutants defining specific defense signaling pathways. These studies established important roles for jasmonic acid (JA) and ethylene (ET) signaling in resistance to *S. sclerotiorum* and identified *A. thaliana coi1* mutants defective in the JA receptor as highly susceptible to this pathogen (Guo and Stotz, 2007). Further mutant studies have subsequently identified a number of additional genes and pathways contributing to *S. sclerotiorum* resistance in *A. thaliana*. These include nitric oxide and reactive oxygen species signaling, auxin signaling components, the ABCG40 transporter, the *AN* (*ANGUSTIFOLIA*) gene modulating JA biosynthesis, the GDSL1 lipase, and camalexin and aliphatic glucosinolate metabolites (Perchepied et al., 2010; Stotz et al., 2011a, Stotz et al., 2011b; Ding et al., 2020; Sucher et al., 2020; Gao et al., 2021). Additionally, a proteinaceous elicitor called SCFE1 (SCLEROTINIA CULTURE FILTRATE ELICITOR) was identified that induces responses similar to microbe-associated molecular patterns (MAMPs) from other pathogens (Zhang et al., 2013a). Several *A. thaliana* ecotypes exhibited insensitivity to this elicitor, allowing for mapping of the *RLP30* gene encoding a receptor-like protein required for perception of SCFE1. Knockout mutants for *RLP30* as well as the *BAK1* (*BRASSINOSTEROID-INSENSITIVE1-ASSOCIATED RECEPTOR KINASE1)* and *SOBIR1 (SUPPRESSOR OF BIR1-1)* components involved in MAMP signaling exhibited increased susceptibility to *S. sclerotiorum* (Zhang et al., 2013a).

In addition to the genes and pathways described above, the MED16 subunit of the *A. thaliana* transcriptional Mediator complex has also been implicated in resistance to *S. sclerotiorum* (Wang et al., 2015). Mediator is a large, multi-subunit protein complex conserved among eukaryotes that serves as a transcriptional cofactor to RNA polymerase II and integrates regulatory information through interactions with gene-specific as well as general transcription factors (Kidd et al., 2011; An and Mou, 2013; Chen et al., 2022). Numerous Mediator complex subunits have been implicated in *A. thaliana* defense responses against a variety of pathogens with different infection strategies. Loss-of-function *med16* mutants were highly susceptible to *S. sclerotiorum* and more susceptible than the JA signaling defective *coi1* mutant, indicating a specific role for this subunit in response to *S. sclerotiorum* (Wang et al., 2015). The *A. thaliana* MED15 and MED16 Mediator subunits were found to play a role in SA signaling and activation of systemic acquired resistance (SAR) and *med16* mutants also exhibited increased susceptibility to the necrotrophic pathogens *Alternaria brassicicola* and *Botrytis cinerea* (Wathagula et al., 2012; Zhang et al., 2012). Similarly, *med21* and *med25* mutants also exhibited increased susceptibility to *A. brassicicola* and *B. cinerea* and *med8* mutants are also hypersusceptible to *B. cinerea* (Dhawan et al., 2009; Kidd et al., 2009; Li et al., 2018). The MED14 Mediator subunit was also found to be required for activation of SAR, and mutants exhibited increased susceptibility to virulent and avirulent strains of the bacterial pathogen *Pseudomonas syringae* pv. *tomato* DC3000 (Zhang et al., 2013b). In contrast to responses observed for necrotrophic pathogens, *A. thaliana med8, med18, med20a*, and *med25* mutants exhibit increased resistance to the hemibiotrophic fungus *Fusarium oxysporum* (Kidd et al., 2009; Fallath et al., 2017). Collectively, these results highlight the central role of the Mediator complex in integrating transcriptional signals relating to pathogen defense activation and indicate that specific subunits may play distinct roles in response to pathogens with different infection strategies.

Studies of mutants have been important in establishing roles for specific signaling pathways in resistance to *S. sclerotiorum*; however, whether relevant natural variation in these genes contributes to differences in response among *A. thaliana* accessions has not been established. Although biotechnological approaches for altering crop traits have proliferated and will be further advanced using CRISPR gene editing technology, breeding of most crop species still relies primarily or exclusively on naturally existing genetic variation. Thus, it is of considerable value to understand the natural variation driving intraspecific differences in resistance to this fungus. More recent research has utilized genome-wide association studies (GWAS) involving naturally occurring *A. thaliana* ecotypes to map loci contributing to *S. sclerotiorum* resistance. GWAS carried out using 84 *A. thaliana* ecotypes inoculated with *S. sclerotiorum* isolate 1980 identified two significant associations for resistance, and a prolyl-oligopeptidase was found to underly an association with resistance on chromosome 1 (Badet et al., 2017). In a follow-up study involving 100 *A. thaliana* ecotypes, expression variation associated with polymorphisms in the promoter region of the *ARPC4* gene encoding an actin-related protein contributing to cytoskeletal dynamics was associated with resistance to *S. sclerotiorum* (Badet et al., 2019). Additionally, automated phenotyping of disease development on six *A. thaliana* ecotypes inoculated with seven different *S. sclerotiorum* isolates identified the *LAZ5* disease resistance gene encoding a canonical nucleotide-binding leucine-rich repeat (NB-LRR) resistance protein as a likely susceptibility factor, with mutants in this gene exhibiting enhanced resistance to *S. sclerotiorum* (Barbacci et al., 2020).

Accumulating evidence indicates that different plant genotypes within host species do not necessarily respond similarly to distinct *S. sclerotiorum* isolates. Significant plant genotype × pathogen isolate interactions have been reported for soybean, canola, and sunflower diseases caused by *S. sclerotiorum* (Garg et al., 2010; Davar et al., 2011; Ge et al., 2012; Willbur et al., 2017; Musa-Khalifani et al., 2021; Buchwaldt et al., 2022; Angidi et al., 2025). Consistent with these studies in crop hosts, significant genotype × isolate interactions were also observed in a study of 17 *A. thaliana* ecotypes inoculated with 8 diverse *S. sclerotiorum* isolates (Ge and Barbetti, 2019). Although it is increasingly clear that genetic diversity among *S. sclerotiorum* isolates plays an important role in determining the outcome of interactions with distinct host genotypes, most studies delineating genes and molecular mechanisms involved in resistance to *S. sclerotiorum* have involved single isolates of the pathogen.

Given the need for additional information on natural variation underlying differences in resistance to *S. sclerotiorum* and the emerging need to incorporate *S. sclerotiorum* isolate diversity into such genetic studies, the present study utilized GWAS of *A. thaliana* responses to *S. sclerotiorum* using a larger number of plant genotypes, two timepoints after inoculation, and two pathogen isolates differing in aggressiveness. We identified a total of 30 loci significantly associated with resistance to at least one isolate at one of the timepoints evaluated. Strikingly, responses of the 315 *A. thaliana* ecotypes evaluated for resistance to both *S. sclerotiorum* isolates were relatively weakly correlated, and no overlapping loci were mapped for resistance to the two isolates. Follow-up studies focused on a single mapped locus with multiple potential candidate genes identified a contribution of the Mediator complex subunit MED20a to *S. sclerotiorum* resistance, and variation in *MED20a* expression patterns was observed in susceptible compared with partially resistant ecotypes. These results expand knowledge of mechanisms underlying quantitative resistance to *S. sclerotiorum* and will inform future efforts aimed at genetic dissection of resistance to this destructive pathogen.

## Materials and methods

### Plant materials and growth conditions

Transferred DNA (T-DNA) insertional mutant lines of *Arabidopsis thaliana* (L.) Heynh. with insertions in genes At2g28210 (SALK_080341C), At2g28220 (SALK_026063C), and At2g28225 (SALK_012867C), along with a set of 359 natural accessions (stock # CS76309; Li et al., 2010), were acquired from the Arabidopsis Biological Resource Center (Ohio State University, Columbus, OH). The *med20a* (At2g28230) mutant was kindly provided by Dr. Yun Ju Kim (Kim et al., 2011). *A. thaliana* plants were grown in growth chambers at 21 °C with a 16-h photoperiod and a light intensity of 120 µM m^−2^ s^−1^.

### *S. sclerotiorum* isolates, growth conditions, and inoculum preparation

*S. sclerotiorum* isolates 1980, BN325, BN500, JS558, SunA320, and NE-Canola-07 were collected from *Phaseolus vulgaris*, *Lactuca sativa*, *Brassica napus*, *P. vulgaris*, *Helianthus annuus*, and *B. napus*, respectively, and have been described previously (Amselem et al., 2011; Otto-Hanson et al., 2011; Aldrich-Wolfe et al., 2015; Poudel et al., 2023). Isolates were initially grown by plating a single sclerotium on potato dextrose agar (PDA) and incubating at 22°C for 4 days. Ground mycelial inoculum was prepared by excising three 5-mm plugs from the edge of the growing colony using a cork borer and transferring to a 50-ml conical tube containing 25 ml of potato dextrose broth (PDB). The PDB liquid culture was incubated on a platform shaker at 22°C with shaking at 90 rpm for 3 days. The mycelial culture was then homogenized using a Polytron homogenizer (Kinematica) with speed setting three for 25 s. The homogenized mycelial inoculum was subsequently evaluated using a spectrophotometer (BioPhotometer, Eppendorf) and adjusted to an optical density of 0.5 at 700-nm wavelength.

### Plant inoculations and disease evaluations

Prior to inoculation with *S. sclerotiorum*, *A. thaliana* plants were grown for 3 weeks in a growth chamber as described above. Three-week old plants were inoculated by spotting 10 µl of ground mycelial inoculum onto two leaves per plant near the leaf tip, avoiding the mid-vein. Inoculated plants were then covered with a humidity dome, and the growth chamber temperature was reduced to 18°C to compensate for the increased temperature beneath the humidity dome. Plants were visually evaluated for disease at 4 and 7 days post inoculation (dpi) using the disease rating scale shown in **Table 1**. For initial evaluations of *S. sclerotiorum* isolate aggressiveness, six *S. sclerotiorum* isolates were inoculated on *A. thaliana* ecotype Col-0. A total of six plants were inoculated with each *S. sclerotiorum* isolate, and the experiment was arranged in a randomized complete block design. Disease ratings at 4 and 7 dpi were analyzed using a generalized linear mixed model (GLMM) implemented in SAS version 9.4 (PROC GLIMMIX) with block considered as a random effect. For analysis of ordinal rating data, the GLMM was fit with a multinomial distribution and cumulative logit link function. Means were separated using Tukey’s *post-hoc* test at α = 0.05 (Tukey, 1949). The experiment was repeated with similar results. To facilitate genome-wide association studies, 325 *A. thaliana* ecotypes were evaluated for response to the weakly aggressive *S. sclerotiorum* isolate BN325 and the highly aggressive isolate 1980. Disease evaluations of 325 *A. thaliana* ecotypes were carried out in α-lattice designs in which each of the two isolates was inoculated onto nine plants of each *A. thaliana* ecotype. Disease ratings at 4 and 7 dpi from α-lattice experiments were analyzed using a GLMM fit with a multinomial distribution and cumulative logit link function with replicate and block (within replicate) considered as random effects. Means were separated using Tukey’s *post-hoc* test at α = 0.05. For evaluation of *A. thaliana* mutant lines, 15 plants of each mutant line, along with the parent line Col-0, were inoculated with *S. sclerotiorum* isolates BN325 or 1980 in each experimental run and three independent experimental runs were conducted. Disease rating data for the three experimental runs was evaluated for homogeneity of variance across experiments using the Fligner-Killeen test via the fligner.test function of R v. 4.3.2 (Fligner and Killeen, 1976). The data from three experimental runs were combined for further analysis upon confirmation of homogeneity of variance. Disease rating data were then evaluated using a GLMM as described above considering experimental run and replicate (within experimental run) as random effects. Means were compared with the Col-0 parent line using Dunnett’s test at α = 0.05 (Dunnett, 1955).

**Table 1:** Rating scale used to score *S. sclerotiorum* disease severity on *A. thaliana* ecotypes at 4- and 7-days post inoculation by spotting of leaves with ground mycelia.

Score	Observation
0	No visible lesion
1	Lesion confined to inoculation site
2	Lesion <25% inoculated leaf area
3	Lesion 25%-50% inoculated leaf area
4	Lesion 50%-75% inoculated leaf area
5	Lesion 75%-100% inoculated leaf area
6	Lesion beyond inoculated leaf but >25% plant area
7	Lesion 25%-50% plant area
8	Lesion 50%-75% plant area
9	Lesion 75%-100% plant area

### Genome-wide association studies

Publicly available imputed single-nucleotide polymorphism (SNP) data for 2029 A*. thaliana* accessions were obtained (Arouisse et al., 2020; https://figshare.com/articles/dataset/arabidopsis_2029_Maf001_filter95/11346875/1). These SNP data were filtered to retain only the 325 ecotypes used in this study and to remove markers with minor allele frequency <3% and missing data >10%. The filtered SNP dataset was used with disease rating data for 4 and 7 dpi with *S. sclerotiorum* isolates BN325 and 1980 to carry out genome-wide association studies (GWAS) using the FarmCPU (fixed and random model circulating probability unity) algorithm in the R package GAPIT v. 3 where population structure correction was based on three principal components (Wang and Zhang, 2021). GWAS was also carried out using GEMMA (genome-wide efficient mixed model association; Zhou and Stephens, 2012). Genome-wide significance thresholds for associated markers were determined using the Bonferroni correction as well as the SimpleM method (Gao et al., 2008). Model fits were examined using quantile–quantile (QQ) plots (**Supplementary Figures S2**, **S3**). Linkage disequilibrium (LD) blocks surrounding associated SNP markers passing the SimpleM significance threshold were determined using PLINK v. 1.9 with an R^2^ value of 0.6 to define LD (Purcell et al., 2007). Annotations for genes within LD blocks surrounding significant associations were obtained from TAIR (The Arabidopsis Information Resource; www.arabidopsis.org).

### *MED20a* sequencing

Sequencing of the *MED20a* (At2g28230) genomic region including 1,500 bp upstream of the start codon and spanning the 3′ untranslated region was carried out using primers listed in **Supplementary Table S2**. DNA was isolated from *A. thaliana* ecotypes Col-0, Bg-2, Lm-2, Shahdara, TOU-I-17, Ag-0, Dra-2, UKSE06-414, and Zdr-6 using a GeneJET Plant Genomic DNA purification kit (Thermo Fisher Scientific) according to the manufacturer’s protocol. DNA concentrations were assessed using a Qubit 3.0 fluorometer. Polymerase chain reaction (PCR) fragments were generated using primer pairs *MED20a* Fwd-1/*MED20a* Rev-1 through *MED20a* Fwd-6/*MED20a* Rev-6. PCR was carried out in 25-µl reaction volumes containing 50 ng genomic DNA, 10 µM of each primer, 2.5 U of Taq DNA polymerase (Accuris Life Science Reagents), 1× Taq reaction buffer, and 100 mM dNTPs. PCR reactions were subjected to thermal cycling with an initial denaturation of 94°C for 3 min; 35 cycles of 94°C for 30 s, annealing for 30 s at 50°C to 54°C depending on primer pair; and 72°C for 1 min, with a final extension at 72°C for 10 min. Aliquots of 5 µl were electrophoresed on 1% agarose gels to verify amplification, and PCR products were cleaned up using ExoSAP-IT reagent (Thermo Fisher Scientific). PCR products were sequenced using Sanger technology by Eurofins Genomics with sequencing from both ends of each PCR product in separate reactions. Multiple sequence alignments were performed using Clustal Omega (Sievers et al., 2011).

### Real-time quantitative PCR

Total RNA was isolated from leaf tissues of *A. thaliana* ecotypes Ag-0, Dra-2, UKSE06-414, Zdr-6, Bg-2, Lm-2, Shahdara, and TOU-I-17 collected at 0, 8, 24, and 48 h post inoculation (hpi) with *S. sclerotiorum* isolate BN325 using a GeneJET Plant RNA isolation kit (Thermo Fisher Scientific). A total of 500 ng of RNA for each sample was treated with DNase I and used in a reverse transcription reaction with iScript Reverse Transcription Supermix (Bio-Rad) according to the manufacturer’s instructions. RT-qPCR was performed using 1 µl cDNA, SsoAdvanced Universal SYBR Green Supermix (Bio-Rad), and primers to amplify a region of the *MED20a* transcript (fwd: GTTGCCGGGTAAGTTTGTGA; rev: CAGCTGTGTGTTGTGGAGTG) or to amplify a region of the *UBQ10* transcript as an internal standard (fwd: GGCCTTGTATAATCCCTGATGAATAAG; rev: AAAGAGATAACAGGAACGGAAACATAGT). A Bio-Rad CFX Connect instrument (Bio-Rad) was used to perform cycling with a program of 50°C for 2 min; 95°C for 2 min; and 40 cycles of 95°C for 15 s, 58°C for 15 s, and 72°C for 1 min, with data collection at the end of each cycle. Data were analyzed using the ΔΔC_T_ method (Schmittgen and Livak, 2008) with CFX Manager software (Bio-Rad). *UBQ10* was used as the internal control to normalize all samples, and the 0-hpi timepoint was used as the reference sample for each ecotype.

## Results

### Responses of 325 *A. thaliana* ecotypes to inoculation with two *S. sclerotiorum* isolates differing in aggressiveness

**Figure 1:**
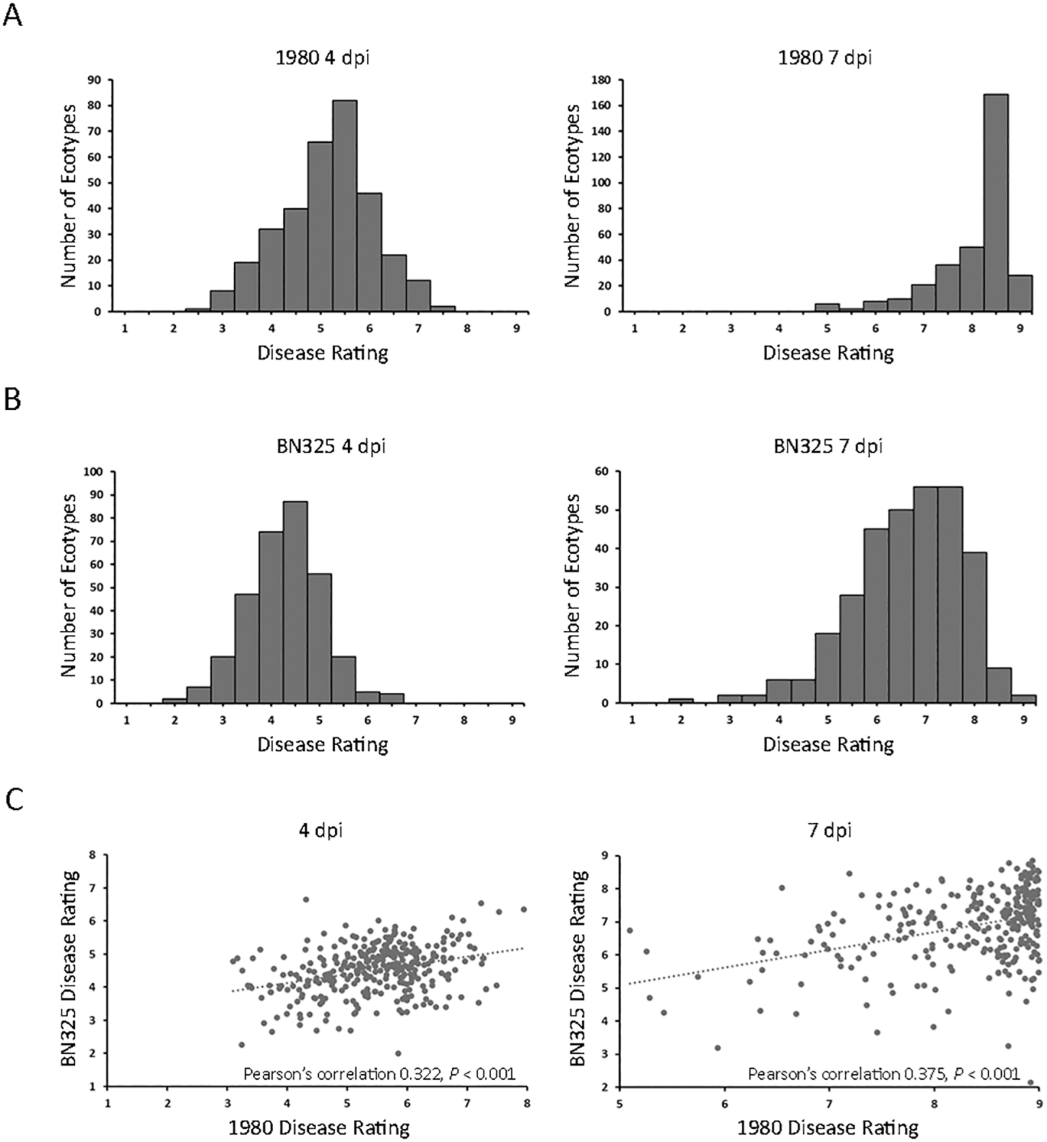
Disease responses of 325 *A. thaliana* ecotypes inoculated with two *S. sclerotiorum* isolates. **(A)** Distributions of mean disease rating scores for 325 *A. thaliana* ecotypes at 4 days post inoculation (dpi; left panel) and 7 dpi (right panel) with *S. sclerotiorum* isolate 1980. **(B)** Distributions of mean disease rating scores for 315 *A. thaliana* ecotypes at 4 dpi (left panel) and 7 dpi (right panel) with *S. sclerotiorum* isolate BN325. **(C)** Scatterplots of disease ratings of 315 *A. thaliana* ecotypes at 4 dpi (left panel) and 7 dpi (right panel) with *S. sclerotiorum* isolates 1980 (x-axis) and BN 325 (y-axis) and Pearson’s correlation coefficients.

A collection of 325 geographically and genetically diverse *A. thaliana* ecotypes was evaluated for response to the aggressive and fully sequenced reference *S. sclerotiorum* isolate 1980. Disease progression was scored at both 4 and 7 dpi as the 4-dpi timepoint generally reflected the timing at which the expanding lesion formed by isolate 1980 on *A. thaliana* ecotype Col-0 encompassed the entire inoculated leaf and began to move into the petiole (**Table 1**). We reasoned that different defense mechanisms may operate in resistance to lesion expansion within the original inoculated leaf compared with those potentially involved in restricting lesion expansion into the petiole and systemically throughout the rest of plant. Significant differences (*P* < 0.001) in levels of resistance to *S. sclerotiorum* isolate 1980 were observed between *A. thaliana* ecotypes at both the 4- and 7-dpi timepoints. At 4 dpi, mean disease scores ranged from 2.98 for ecotype Fja1–1 to 7.95 for ecotype Wa-1, reflecting a very broad range of responses to inoculation in which the most resistant ecotype exhibited small lesions that encompassed <50% of the inoculated leaf area whereas the most susceptible ecotype exhibited lesions that covered over 50% of the entire plant area (**Table 1**; **Figure 1A**; **Supplementary Table S1**). At 7 dpi, mean disease scores ranged from 5.01 for ecotype Tad01 to the highest possible disease score of 9, observed for 28 ecotypes and reflecting expansion of the disease lesion to cover the entire plant area resulting in plant death (**Figure 1A**; **Supplementary Table S1**). At 7 dpi with isolate 1980, the majority of *A. thaliana* ecotypes exhibited severe disease with lesions covering over 75% of the plant area (**Figure 1A**).

To determine if *A. thaliana* ecotypes responded similarly to different *S. sclerotiorum* isolates, we first sought to identify an isolate exhibiting a significantly different level of aggressiveness on *A. thaliana* compared with isolate 1980. A total of six *S. sclerotiorum* isolates, including isolate 1980, collected from different host plants were inoculated onto *A. thaliana* ecotype Col-0, and disease was evaluated at 4 and 7 dpi. Isolate JS558 collected from *P. vulgaris* in Minnesota was significantly more aggressive than isolate 1980 on Col-0 at both timepoints whereas isolate BN325, collected from *L. sativa* in Arizona, was significantly less aggressive than 1980 at both timepoints (**Supplementary Figure S1**). Given that isolate 1980 caused severe disease on most tested *A. thaliana* ecotypes at 7 dpi, we selected the weakly aggressive isolate BN325 for additional phenotyping of the *A. thaliana* ecotype collection. A total of 315 of the 325 *A. thaliana* ecotypes were evaluated for response to *S. sclerotiorum* isolate BN325 whereas 10 ecotypes were omitted due to limited seed availability. Significant differences (*P* < 0.001) in response to isolate BN325 were observed between ecotypes at both 4 and 7 dpi. At 4 dpi with BN325, mean disease scores ranged from 2.01 for ecotype Dra-2 to 6.65 for ecotype Ler-1 (**Figure 1B**; **Supplementary Table S1**). At 7 dpi, mean disease scores ranged from 2.75 for ecotype Sei-0 to the highest possible disease score of 9 for ecotypes Pro-0 and Bg-2 (**Figure 1B**; **Supplementary Table S1**). Consistent with initial observations on ecotype Col-0, isolate BN325 was generally less aggressive than isolate 1980 on *A. thaliana*, with lower disease scores at both 4 and 7 dpi and only two ecotypes exhibiting complete plant death at 7 dpi with BN325 compared with 28 ecotypes at 7 dpi with isolate 1980 (**Figure 1**). Correlations among responses for the 315 ecotypes inoculated with both isolates were evaluated for both timepoints. At 4 dpi, a significant correlation (*P* < 0.001) was observed for ecotype responses to the two isolates with Pearson’s correlation coefficient of 0.322, whereas at 7 dpi, a similarly significant correlation (*P* < 0.001) was observed with Pearson’s correlation coefficient of 0.375 (**Figure 1C**).

### Genome-wide association mapping of *A. thaliana* loci contributing to *S. sclerotiorum* resistance

**Figure 2:**
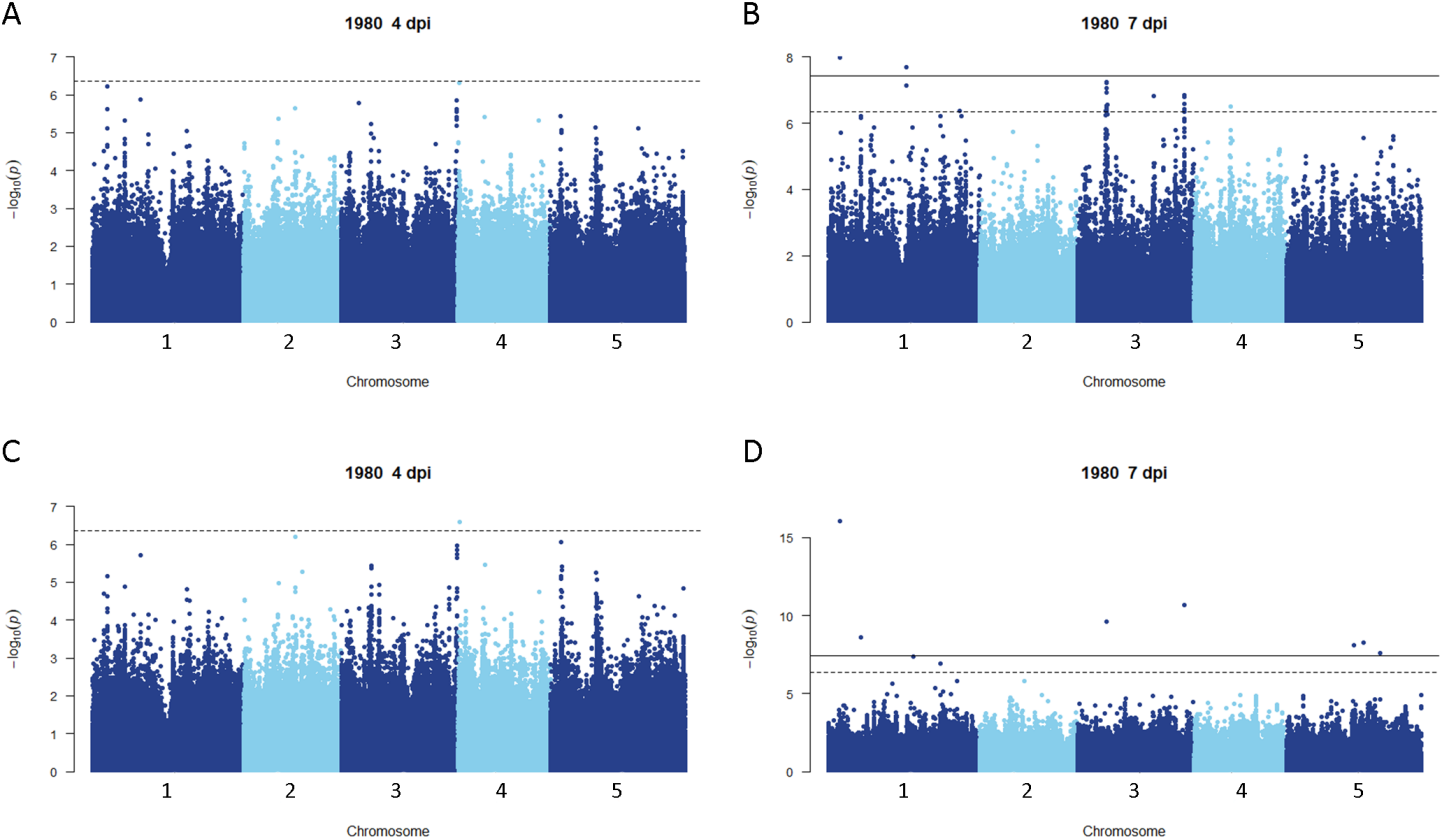
Genome-wide association for *A. thaliana* resistance to *S. sclerotiorum* isolate 1980. **(A, B)** Manhattan plots of genome-wide association for *A. thaliana* resistance to *S. sclerotiorum* isolate 1980 at 4 and 7 dpi determined using GEMMA. **(C, D)** Manhattan plots of genome-wide association for *A. thaliana* resistance to *S. sclerotiorum* isolate 1980 at 4 and 7 dpi determined using FarmCPU. In all panels, solid lines indicate genome-wide significance thresholds determined using the Bonferroni multiple comparison correction, and dashed lines indicate significance thresholds determined using the SimpleM method.

**Figure 3:**
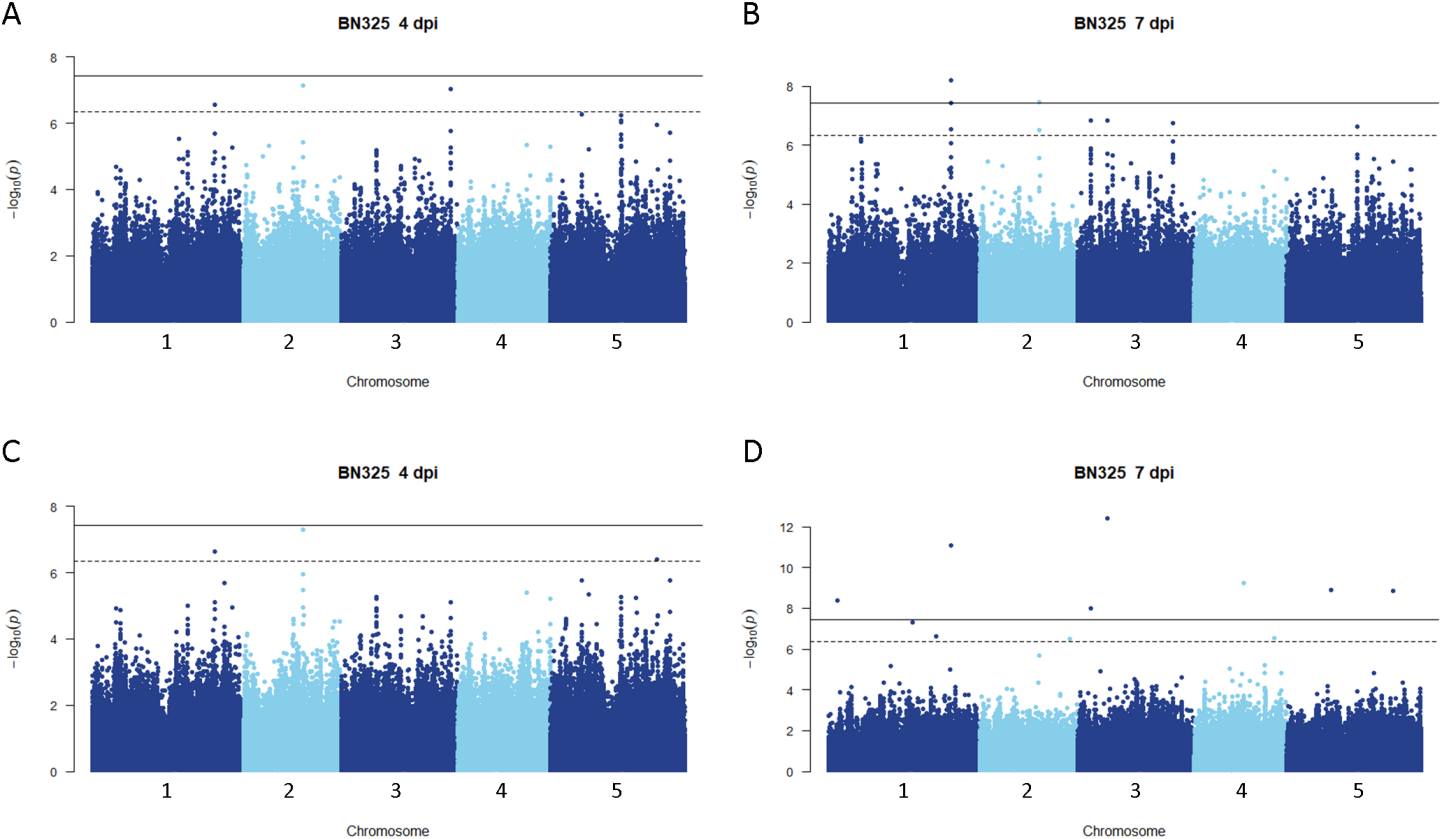
Genome-wide association for *A. thaliana* resistance to *S. sclerotiorum* isolate BN325. **(A, B)** Manhattan plots of genome-wide association for *A. thaliana* resistance to *S. sclerotiorum* isolate BN325 at 4 and 7 dpi determined using GEMMA **(C, D)** Manhattan plots of genome-wide association for *A. thaliana* resistance to *S. sclerotiorum* isolate BN325 at 4 and 7 dpi determined using FarmCPU. In all panels, solid lines indicate genome-wide significance thresholds determined using the Bonferroni multiple comparison correction, and dashed lines indicate significance thresholds determined using the SimpleM method.

Disease rating data at 4 and 7 dpi for the *A. thaliana* ecotype collection inoculated with *S. sclerotiorum* isolates 1980 or BN325 were used along with SNP genotyping data from a publicly available dataset to carry out genome-wide association studies (GWAS) of resistance to the two isolates. Two different models, GEMMA and FarmCPU, were used to carry out GWAS, and results from both models are reported. Associations were named Qrss for quantitative trait locus (QTL) for resistance to *S. sclerotiorum*, followed by the *A. thaliana* chromosome number and the number of the QTL on that chromosome. For resistance to isolate 1980 at the 4-dpi timepoint, only a single significant association was identified on chromosome 4 by both the GEMMA and FarmCPU models (**Figure 2**; **Table 2**; **Supplementary Figure S2**). A total of four significant associations were identified for resistance to isolate 1980 at the 7-dpi timepoint by the GEMMA model, whereas six significant associations were identified by the FarmCPU model and three associations were identified by both models (**Figure 2**; **Table 2**; **Supplementary Figure S2**). SNP markers significantly associated with resistance to isolate 1980 at the 7-dpi timepoint were found on *A. thaliana* chromosomes 1, 3, 4, and 5 (**Figure 2**; **Table 2**). A single association for resistance to isolate BN325 at the 4-dpi timepoint was identified on chromosome 3 by the GEMMA model (**Figure 3**; **Table 2**; **Supplementary Figure S3**). Three significant associations were identified for resistance to isolate BN325 at the 7-dpi timepoint by the GEMMA model, whereas nine significant associations were identified by the FarmCPU model and one association was identified by both models (**Figure 3**; **Table 2**; **Supplementary Figure S3**). Additionally, a single significant association was identified by FarmCPU for resistance to BN325 at both timepoints and two associations were identified by both GWAS models for resistance to BN325 at both timepoints (**Table 2**). SNP markers associated with resistance to isolate BN325 were identified on all five *A. thaliana* chromosomes. Linkage disequilibrium (LD) blocks surrounding each associated SNP marker were defined based on the *A. thaliana* Col-0 reference genome using an R^2^ value of 0.6 as a cutoff. LD blocks varied considerably in their size and the number of genes they encompassed, ranging from a predicted 291-bp LD block lacking any predicted gene models for association Qrss5.4 on chromosome 5 to an ~145-kb LD block encompassing 44 predicted gene models for association Qrss3.3 on chromosome 3 (**Tables 2**; **3**). No common associations nor associations with overlapping LD blocks were identified for resistance to both *S. sclerotiorum* isolates at either timepoint. Annotations for predicted gene models within LD blocks were examined to identify genes with known or predicted functions in defense against pathogens, and annotations for the gene nearest to the associated SNP marker are also presented (**Table 3**). Five associated loci exhibited one or more predicted canonical disease resistance (R) genes with nucleotide binding and leucine-rich repeat domains (NB-LRRs) within the predicted LD blocks, whereas three loci exhibited predicted LRR kinase genes within LD blocks and two loci encompassed WRKY transcription factors (**Table 3**). Of the 30 total associations identified by GWAS, 14 loci did not feature genes with known or predicted defense-related functions within LD of the associated marker (**Table 3**).

**Table 2:** Details for single-nucleotide polymorphism markers associated with resistance to *S. sclerotiorum* isolates 1980 and BN325 discovered by genome-wide association studies.

Association^1^	Isolate	Analysis	*P* ^2^	DPI	Chr	LD Start^3^	Associated SNP	LD End	LD block size
Qrss1.1	BN325	FarmCPU	4.05^-9^	7	1	1891779	1894664	1898590	6811
Qrss1.2	1980	Both	8.29^-17^	7	1	2374616	2381661	2461871	87257
Qrss1.3	1980	FarmCPU	2.47^-9^	7	1	6496653	6509352	6514309	17656
Qrss1.4	1980	GEMMA	1.56^-7^	7	1	15745283	15745810	15746156	873
Qrss1.5	BN325	FarmCPU	4.87^-8^	7	1	16911083	16913718	16916043	4960
Qrss1.6	1980	FarmCPU	4.44^-8^	7	1	17193387	17194651	17208282	14895
Qrss1.7	BN325	FarmCPU	2.33^-7^	7	1	21635569	21637551	21662462	26893
Qrss1.8	1980	FarmCPU	1.17^-7^	7	1	22599097	22601475	22603313	4216
Qrss1.9	BN325	Both	2.30^-7^	Both	1	24622780	24624275	24631450	8670
Qrss1.10	1980	GEMMA	4.16^-7^	7	1	26446971	26447356	26569591	122620
Qrss2.1	BN325	Both	5.16^-8^	Both	2	12006049	12036635	12037690	31641
Qrss2.2	BN325	FarmCPU	3.11^-7^	7	2	18051044	18051183	18068097	17053
Qrss3.1	BN325	FarmCPU	9.73^-9^	7	3	2579107	2588400	2596594	17487
Qrss3.2	BN325	GEMMA	1.46^-7^	7	3	2639267	2643390	2645077	5810
Qrss3.3	1980	Both	2.29^-10^	7	3	5679672	5797124	5825199	145527
Qrss3.4	BN325	Both	1.42^-7^	7	3	6001725	6029308	6046582	44857
Qrss3.5	1980	GEMMA	1.56^-7^	7	3	15359873	15377530	15411581	51708
Qrss3.6	BN325	GEMMA	1.76^-7^	7	3	19076539	19114860	19129090	52551
Qrss3.7	1980	Both	2.08^-11^	7	3	21404112	21429749	21437028	32916
Qrss3.8	BN325	GEMMA	9.38^-8^	4	3	21959261	21972597	21977336	18075
Qrss4.1	1980	Both	2.60^-7^	4	4	216749	234166	260617	43818
Qrss4.2	1980	GEMMA	3.15^-7^	7	4	7171051	7235338	7237243	66192
Qrss4.3	BN325	FarmCPU	5.68^-10^	7	4	10008067	10008708	10009499	1432
Qrss4.4	BN325	FarmCPU	2.88^-7^	7	4	16054597	16083590	16113835	59238
Qrss5.1	BN325	FarmCPU	1.23^-9^	7	5	8851363	8853147	8855080	3717
Qrss5.2	1980	FarmCPU	8.40^-9^	7	5	13495204	13512917	13570571	75367
Qrss5.3	BN325	GEMMA	2.39^-7^	7	5	14232863	14241562	14275706	42843
Qrss5.4	1980	FarmCPU	5.64^-9^	7	5	15372689	15372792	15372980	291
Qrss5.5	1980	FarmCPU	2.58^-8^	7	5	18738621	18739616	18749628	11007
Qrss5.6	BN325	FarmCPU	1.36^-9^	Both	5	21428860	21429322	21430425	1565

^1^Associations are named Qrss for QTL (quantitative trait locus) for resistance to *Sclerotinia sclerotiorum* followed by chromosome number and QTL number on that chromosome.

^2^Associations with *P* values less than the SimpleM threshold of 4.47^-7^.

^3^LD—linkage disequilibrium based on R^2^ 0.6.

**Table 3:** Candidate genes at loci associated with resistance to *S. sclerotiorum*.

Association	No. of genes in LD^1^	Closest gene	Defense-related genes^2^
Qrss1.1	3	At1g06200 - Peptidase S24/S26A/S26B/S26C family	
Qrss1.2	34	At1g07705 - NOT2/NOT3/NOT5 family	At1g07650 - LRR kinase
Qrss1.3	2	At1g18860 - WRKY61	At1g18860 - WRKY61; At1g18870 - ICS2
Qrss1.4	1	At1g42150 - transposable element gene	
Qrss1.5	3	At1g44790 - ChaC-like family protein	
Qrss1.6	4	At1g45760 - transposable element gene	At1g45616 - RLP6
Qrss1.7	2	At1g58330 - transcription factor-like protein	
Qrss1.8	1	At1g61270 - Transmembrane amino acid transporter family	At1g61300 - NB-LRR; At1g61310 - NB-LRR
Qrss1.9	2	At1g66140 - ZFP4 (zinc finger protein 4)	At1g660909 - NB-LRR
Qrss1.10	27	At1g70220 - RNA processing Lsm domain-containing protein	At1g70520 - CRK2; AT1g70530 - CRK3
Qrss2.1	8	At2g28225 - aspartic protease CDR1-like protein	At2g28220 - CDR1-like; At2g28225 - CDR1-like; At2g28230 - MED20a
Qrss2.2	5	At2g43470 - Protein of unknown function (DUF3755)	At2g43410 - FPA
Qrss3.1	5	At3g08530 - CHC2 (clathrin heavy chain 2)	
Qrss3.2	3	At3g08700 - ubiquitin-conjugating enzyme 12	At3g08680 - LRR kinase
Qrss3.3	44	At3g17000 - ubiquitin conjugating enzyme 32	
Qrss3.4	19	At3g17630 - AtCHX19 (cation/H+ exchanger 19)	
Qrss3.5	19	At3g43444 - transposable element gene	At3g43440 - JAZ11 (jasmonate zim domain protein 11)
Qrss3.6	19	At3g51530 - F-box/RNI-like/FBD-like domains containing protein	At3g51550 -FER (Feronia); At3g51560 - NB-LRR; At3g51570 - NB-LRR
Qrss3.7	11	At3g57870 - SCE1 (sumo conjugation enzyme 1)	At3g57830 - LRR kinase
Qrss3.8	5	At3g59450 - calcium-binding EF hand family protein	
Qrss4.1	15	At4g00540 - myb domain protein 3r2	
Qrss4.2	14	At4g12070 - hypothetical protein	At4g12010 - NB-LRR
Qrss4.3	0	At4g18020 - APRR2 (pseudo-response regulator 2)	
Qrss4.4	5	At4g33410 - SPPL1 (signal peptide peptidase-like 1)	At4g33430 - BAK1
Qrss5.1	2	At5g25430 - HCO3 transporter family	
Qrss5.2	19	At5g35280 - transposable element gene	
Qrss5.3	12	At5g36180 - SCPL1 (serine carboxypeptidase-like 1)	
Qrss5.4	0	At5g38396 - F-box/RNI-like superfamily protein	
Qrss5.5	3	At5g46220 - TOD1 (turgor regulation defect 1)	At5g46260 - NB-LRR; At5g46270 - NB-LRR
Qrss5.6	1	At5g52870 - MAKR5 (membrane-associated kinase regulator 5)	At5g52830 - WRKY27

^1^LD—linkage disequilibrium based on R^2^ 0.6.

^2^Defense-related genes determined based on TAIR annotations and published literature.

### *A. thaliana* mediator complex subunit MED20a contributes to resistance against *S. sclerotiorum*

**Figure 4:**
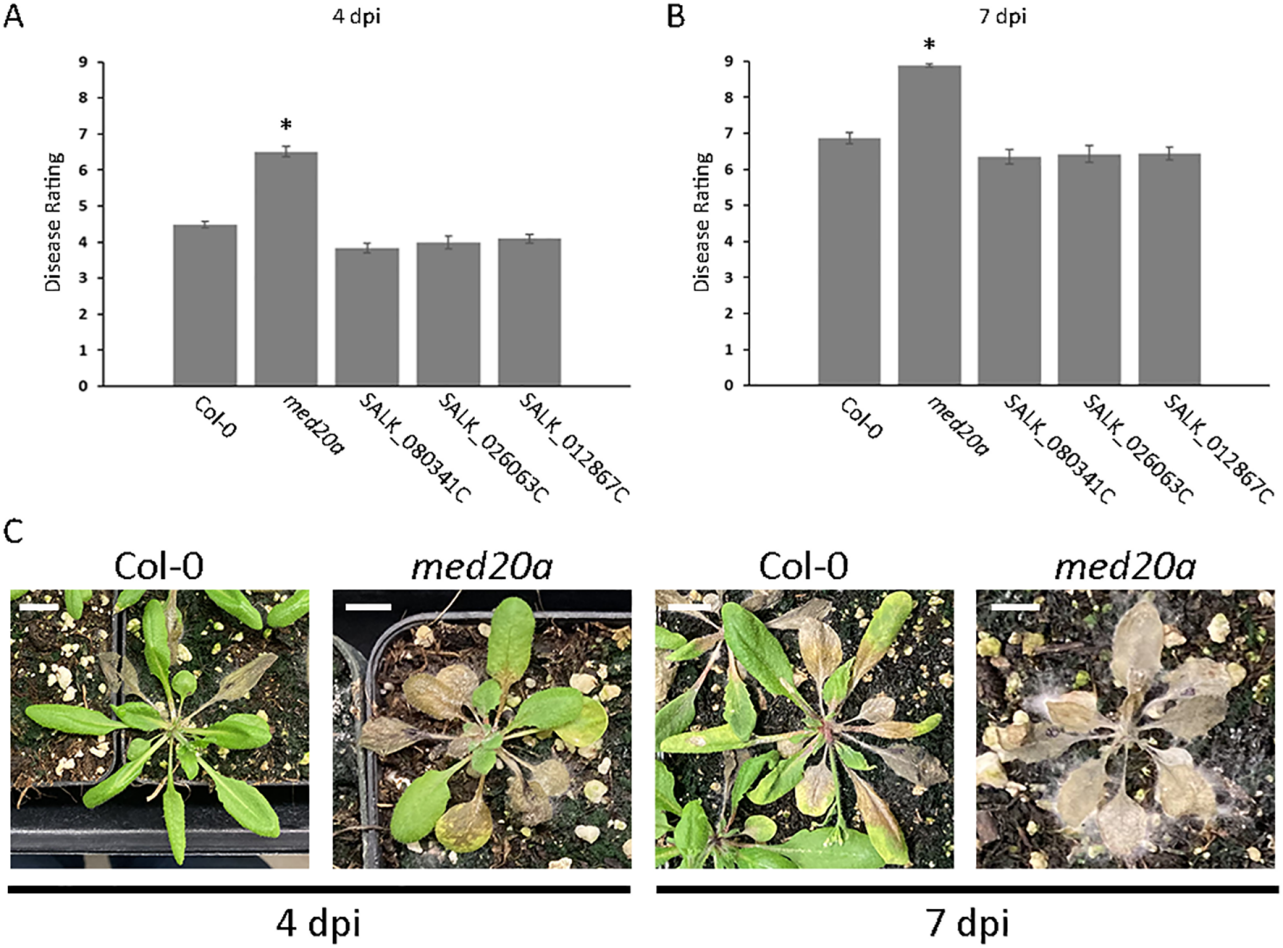
Hypersusceptibility of the *A. thaliana med20a* mutant to *S. sclerotiorum.***(A, B)** Plots of mean disease ratings for the parent ecotype Col-0, the *med20a* (At2g28230) mutant, and T-DNA insertion lines SALK_080341C (At2g28210), SALK_026063C (At2g28220), and SALK_012867C (At2g28225) at 4 and 7 dpi with *S. sclerotiorum* isolate BN325. Error bars indicate SEM (n = 45). Asterisks indicate statistically significant differences compared with Col-0 (generalized linear mixed model and Dunnett’s test, *P* < 0.01). **(C)** Representative images illustrating disease progression on *A. thaliana* ecotype Col-0 and the *med20a* mutant at 4 and 7 dpi with *S. sclerotiorum* isolate BN325. Scale bars: 10 mm.

Association Qrss2.1 on *A. thaliana* chromosome 2 was identified by both GWAS models at both timepoints for resistance to *S. sclerotiorum* isolate BN325 (**Table 2**). This associated locus encompasses an LD block of ~32 kb containing eight predicted gene models (**Tables 2**, **3**). Two predicted genes with similarity to the *CONSTITUTIVE DISEASE RESISTANCE1* (*CDR1*) gene encoding an aspartic protease involved in disease resistance lie near the associated marker (Xia et al., 2004). Additionally, the *MED20a* gene encoding a subunit of the transcriptional Mediator complex also lies within the Qrss2.1 LD block near the associated marker. Given that there are several potential candidate genes at this locus and that another *A. thaliana* Mediator complex subunit, MED16, has previously been implicated in resistance to *S. sclerotiorum*, we selected Qrss2.1 for further study to determine if the gene underlying resistance could be identified (Wang et al., 2015). Four predicted candidate genes near the associated marker at Qrss2.1 were prioritized to determine if one or more of these genes is required for full resistance to *S. sclerotiorum*. As noted above, candidate genes At2g28220 and At2g28225 exhibit similarity to the defense-related gene *CDR1*. T-DNA (transferred DNA) insertional mutants SALK_026063C, carrying an insertion in the single predicted exon of At2g28220, and SALK_012867C, carrying an insertion in the single predicted exon of At2g28225, were obtained to evaluate the potential role of these candidate genes in *S. sclerotiorum* resistance. Candidate gene At2g28210 encodes the αCA2 alpha carbonic anhydrase, and a T-DNA insertional mutant, SALK_080341C, carrying an insertion in the second exon of this gene was acquired to evaluate its potential role in resistance to *S. sclerotiorum*. Finally, At2g28230 encodes the MED20a subunit of the transcriptional Mediator complex and a *med20a* mutant carrying a single base substitution in the second exon resulting in a premature stop codon was obtained (Kim et al., 2011). The disease responses of SALK_080341C, SALK_026062C, and SALK_12867C were not significantly different than the Col-0 parent line at 4 or 7 dpi with *S. sclerotiorum* isolate BN325 (**Figures 4A, B**). In contrast, *med20a* mutants were significantly more susceptible to *S. sclerotiorum* isolate BN325 at both 4 and 7 dpi compared with Col-0 (**Figures 4A–C**). At 4 dpi, the disease lesions caused by isolate BN325 had typically encompassed only the inoculated leaf on Col-0, whereas the lesions had spread throughout *med20a* mutant plants (**Figure 4C**). At 7 dpi, lesions had spread to encompass ~50%-75% of the plant area for Col-0 whereas *med20a* plants were completely consumed by the disease lesion and died (**Figure 4C**). To determine if increased susceptibility of *med20a* to *S. sclerotiorum* is specific to isolate BN325, disease response of *med20a* was compared with the Col-0 parent line at 4 and 7 dpi with isolate 1980. Similar to results with isolate BN325, the *med20a* mutants were significantly more susceptible to isolate 1980 at both timepoints, indicating that *MED20a* contributes to resistance against both isolates BN325 and 1980 (**Supplementary Figure S4**). These results suggest that *MED20a* is likely to be the gene underlying the significant association with *S. sclerotiorum* resistance at Qrss2.1.

**Figure 5:**
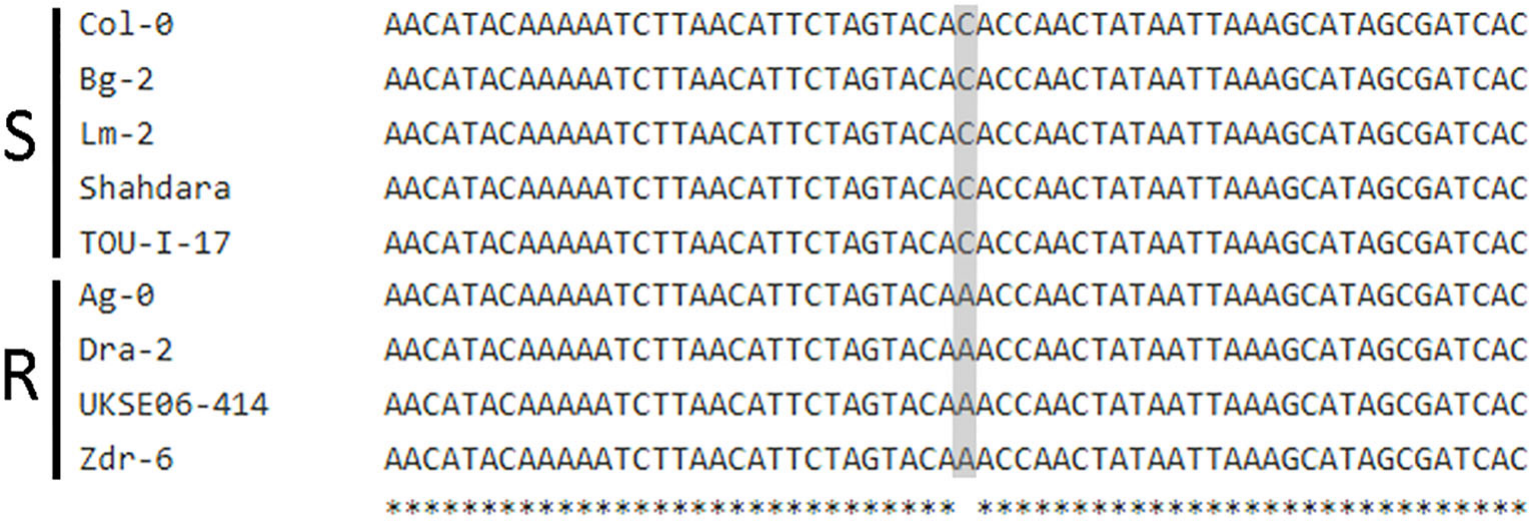
A polymorphism in the *MED20a* promoter region differentiates resistant and susceptible *A. thaliana* ecotypes. Multiple-sequence alignment of sequences for five susceptible (S) and four partially resistant (R) *A. thaliana* ecotypes spanning the region from 1,244 to 1,184 bp upstream of the *Med20a* start codon. Gray shading indicates a C/A polymorphism 1,214 bp upstream of the *Med20a* start codon correlated with the associated marker and resistant or susceptible response.

**Figure 6:**
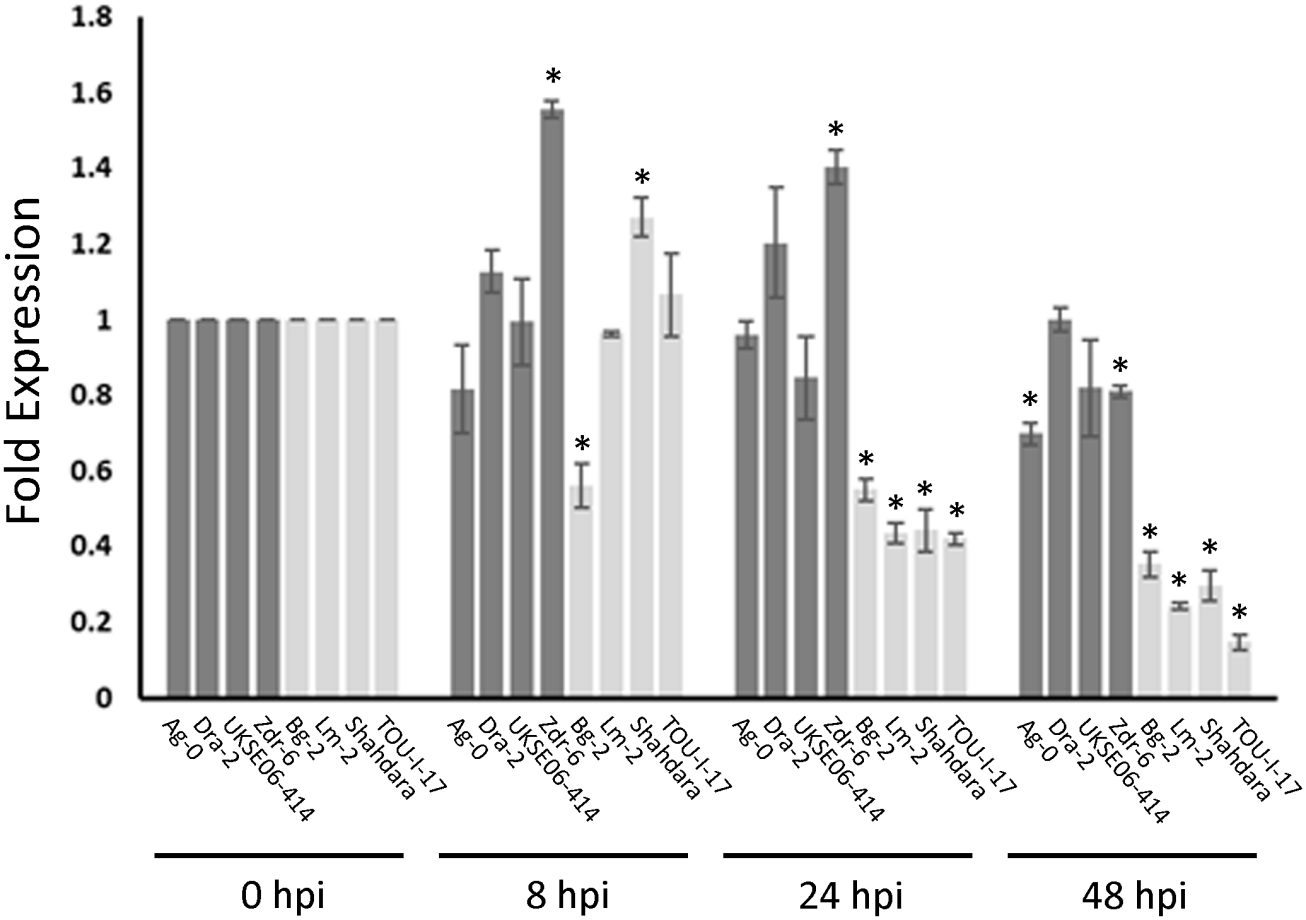
*Med20a* expression in resistant and susceptible ecotypes after *S. sclerotiorum* inoculation. Expression changes at 8, 24, and 48 h post inoculation (hpi) with *S. sclerotiorum* isolate BN325 are depicted as fold expression values relative to expression at 0 hpi for each ecotype. Bars for resistant ecotypes are shaded dark gray, and bars for susceptible ecotypes are shaded light gray. Error bars indicate SEM (n =3), and asterisks indicate significant differences compared with expression at 0 hpi (Student’s T-test, *P* < 0.05).

If *MED20a* is the causal gene affecting *S. sclerotiorum* resistance at Qrss2.1, it would be expected that this gene should possess one or more polymorphisms that co-segregate with the associated marker. To identify such polymorphisms, we sequenced the *MED20a* gene along with ~1,500 bp upstream of the gene start codon in four partially resistant ecotypes that carry the associated marker allele, Ag-0, Dra-2, UKSE06-414, and Zdr-6, and compared the sequences with those from Col-0 and four susceptible ecotypes that do not carry the associated allele: Bg-2, Lm-2, Shahdara, and TOU-I-17. These specific partially resistant ecotypes were chosen because they are among the most resistant ecotypes that carry the associated marker allele at Qrss2.1. Similarly, the susceptible ecotypes were selected because they are among the most susceptible ecotypes observed in the study and they do not carry the associated marker allele at Qrss2.1 Although considerable variation was observed within the coding regions of the gene among the evaluated ecotypes, no polymorphisms in coding regions were found that strictly correlated with the associated marker allele (**Supplementary Figure S5**). When the promoter and regions upstream of the *MED20a* gene were analyzed, an SNP (C/A variant) at 1,214 bp upstream of the *MED20a* start codon was identified that strictly correlates with the associated variant (**Figure 5**). To determine if expression differences are observed in partially resistant ecotypes that carry the resistance-associated allele at Qrss2.1 compared with ecotypes that carry the susceptibility-associated allele, the expression of *MED20a* was evaluated in the above eight ecotypes at 0, 8, 24, and 48 hpi with *S. sclerotiorum* isolate BN325. To compare expression changes after inoculation, expression levels were normalized to the 0-hpi (pre-inoculation) timepoint for each ecotype. At 8 hpi, only three ecotypes exhibited significant differences in expression compared with the 0-hpi timepoint, with partially resistant ecotype Zdr-6 and susceptible ecotype Shahdara exhibiting significantly increased *MED20a* expression and susceptible ecotype Bg-2 exhibiting significantly reduced expression (**Figure 6**). At 24 hpi, clear differences in *MED20a* expression between partially resistant and susceptible ecotypes were observed, with *MED20a* expression remaining elevated in Zdr-6 and unchanged in other partially resistant ecotypes in contrast to significantly reduced *MED20a* expression in all four susceptible ecotypes (**Figure 6**). At 48 hpi, *MED20a* expression levels decreased further in all four susceptible lines whereas expression was not significantly different from 0 hpi for partially resistant ecotypes Dra-2 and UKSE06–414 and was slightly but significantly reduced for ecotypes Ag-0 and Zdr-6 (**Figure 6**).

## Discussion

*S. sclerotiorum* can infect many agriculturally important crop species and, consequently, this pathogen causes considerable damage to global agricultural production. Unlike resistance to biotrophic pathogens that typically can be conferred by single genes, resistance to necrotrophic *S. sclerotiorum* is complex, involving many genes, and no complete resistance has been identified in host species. While studies of defined mutants in the model plant species *A. thaliana* have provided valuable knowledge of specific genes, molecular machinery, and biochemical pathways contributing to *S. sclerotiorum* resistance, information on natural variation underlying intraspecific differences in levels of quantitative resistance to this pathogen is much more limited. Thus, a major goal of this study was to provide a foundation for identifying and defining genes and mechanisms underlying natural variation in resistance to *S. sclerotiorum*. A second major goal was to determine the degree to which *S. sclerotiorum* isolate diversity impacts response to infection among *A. thaliana* ecotypes and influences mapping of loci associated with resistance to the pathogen. We evaluated resistance of over 300 *A. thaliana* ecotypes against two *S. sclerotiorum* isolates that differed significantly in their aggressiveness in causing disease on this host. Somewhat unexpectedly, we observed only weak correlations for resistance among *A. thaliana* ecotypes to the two isolates at both the 4- and 7-dpi timepoints evaluated in the study. These results are consistent with those of a prior study in which responses of some *A. thaliana* ecotypes to infection varied depending on the *S. sclerotiorum* isolate used for inoculation (Ge and Barbetti, 2019). Additionally, significant genotype × isolate interactions have also been observed for *S. sclerotiorum* infection of crop hosts such as soybean, canola, and sunflower (Garg et al., 2010; Davar et al., 2011; Ge et al., 2012; Willbur et al., 2017; Musa-Khalifani et al., 2021; Buchwaldt et al., 2022; Angidi et al., 2025). In contrast to these reports and the results of this study, Barbacci and colleagues reported that differences in responses of *A. thaliana* ecotypes to *S. sclerotiorum* isolates were driven primarily by differences in the latency period between application of inoculum and appearance of an expanding necrotic lesion (Barbacci et al., 2020). Using an automated phenotyping platform, these authors found that lesion expansion after the latency period was primarily determined by *A. thaliana* genotype and only minimally influenced by the *S. sclerotiorum* isolate (Barbacci et al., 2020). If differences in disease response to distinct *S. sclerotiorum* isolates were due primarily to lengths of latency period, we would have expected to see a shift in disease severity but high correlations between responses to different isolates in our study. While we did indeed observe a shift in disease severity in comparing the weakly aggressive isolate BN325 to the more aggressive isolate 1980, we observed only weak correlations among ecotype responses to the two isolates, suggesting that the latency period could not explain the differential responses. The reasons for this discrepancy are not entirely clear, although several explanations are possible. The study from Barbacci and colleagues focused on automated imaging of lesion expansion on detached *A. thaliana* leaves at relatively early timepoints, up to 3 dpi. Thus, this study did not encompass movement of the pathogen beyond the inoculated leaf. Additionally, this study involved only six *A. thaliana* ecotypes and utilized a somewhat different inoculation method. In general, our results provide further support for the observation that responses of host plant genotypes, including *A. thaliana* natural ecotypes, can vary significantly depending on *S. sclerotiorum* isolate.

GWAS were carried out to identify loci contributing to resistance against the two distinct *S. sclerotiorum* isolates at two timepoints after inoculation. The 4- and 7-dpi timepoints were chosen based on observations of disease progression, as the 4-dpi timepoint generally reflects the timing at which *S. sclerotiorum* isolate 1980 begins to move beyond the inoculated leaf on most *A. thaliana* ecotypes, whereas at 7 dpi severe disease is observed in response to this isolate for most ecotypes, with some ecotypes completely consumed by the fungus at this timepoint. Similar total numbers of associated loci were mapped for the two isolates, with 14 associations identified for isolate 1980 across the two timepoints and 16 associations identified for isolate BN325. Strikingly, however, no overlapping loci were mapped for resistance to the two isolates. Although this outcome is consistent with the low correlation between responses to the two isolates among the *A. thaliana* ecotypes evaluated, this was still an unexpected result.

We selected a single associated locus on *A. thaliana* chromosome 2, referred to as Qrss2.1, for validation of the GWAS results and attempted identification of the specific gene underlying variation in resistance. Several potential candidate genes were identified at this locus, including two genes with similarity to the defense-related gene *CDR1* as well as *MED20a* encoding a subunit of the Mediator complex. Evaluation of mutants revealed a role for *MED20a* in resistance to *S. sclerotiorum*, and sequencing identified an SNP in the *MED20a* promoter region that correlated with the associated marker at Qrss2.1 and was associated with differential expression of this gene in partially resistant compared with susceptible ecotypes. Expression polymorphism influencing resistance to *S. sclerotiorum* has also been reported for the actin-related *ARPC4* locus (Badet et al., 2019). These results suggest that the promoter variant upstream of *MED20a* may be the causal polymorphism affecting resistance at Qrss2.1. However, additional research such as modifying this nucleotide in susceptible ecotypes via gene editing approaches will be needed to confirm the effect of this specific variant on resistance to *S. sclerotiorum*. Mutation of *A. thaliana MED20a* has previously been shown to increase resistance to the hemibiotrophic fungus *Fusarium* oxysporum (Fallath et al., 2017). Additionally, Mediator complex subunit MED16 has been implicated in resistance to *S. sclerotiorum* (Zhang et al., 2012). This study reported results for inoculation of a *med20a* mutant with *S. sclerotiorum*, finding no significant impact on resistance. However, the T-DNA insertional mutant used by Zhang and colleagues, GABI_507F08, possesses a T-DNA insertion in the gene At3g28230 rather than in At2g28230 encoding *MED20a*. Thus, a true knockout mutant for *MED20a* had not been previously evaluated for response to *S. sclerotiorum*, explaining this discrepancy. To our knowledge, no previous studies have identified natural variation in genes encoding *A. thaliana* Mediator complex subunits in resistance to *S. sclerotiorum*, as these studies have involved artificially induced mutations. However, GWAS for canola resistance to Sclerotinia stem rot identified an associated polymorphism within the putative canola homolog of *A. thaliana MED15a*, suggesting that natural variation in a Mediator complex subunit may influence resistance to *S. sclerotiorum* in this related species (Newman et al., 2023). The *MED20a* locus was mapped for resistance to *S. sclerotiorum* isolate BN325 at both timepoints in our study. However, *med20a* knockout mutants exhibited hypersusceptibility to both isolate BN325 and isolate 1980, indicating that the contribution of *MED20a* to *S. sclerotiorum* resistance is not specific to isolate BN325. One hypothesis to explain the lack of overlap for mapped loci associated with resistance to the two isolates is that loci involved in resistance may play more or less prominent roles in response to some *S. sclerotiorum* isolates depending on the infection dynamics controlled by isolate genetics. It is also important to note that the *med20a* mutant used in this study is likely a null mutant, whereas the effect of the natural variant in the *MED20a* promoter is almost certainly much less pronounced and this more subtle effect may only be apparent in response to the less aggressive *S. sclerotiorum* isolate. Further research on genes associated with resistance to determine whether or not they contribute to resistance in an isolate-specific manner should help to clarify these observations.

Including *MED20a*, genes with known or predicted functions in pathogen defense were identified at 16 of the 30 total loci mapped across our GWAS. These included five associated loci with one or more predicted NB-LRR disease resistance genes within the LD blocks. The *A. thaliana LAZ5* NB-LRR has previously been identified as a putative susceptibility factor, with mutation in this gene conferring increased resistance to *S. sclerotiorum* (Barbacci et al., 2020). Given the necrotrophic lifestyle of this pathogen, it is reasonable to hypothesize that *S. sclerotiorum* may activate NB-LRR-mediated defenses associated with programmed cell death to promote infection. Whether the NB-LRRs located within mapped loci in our study also act as susceptibility factors exploited by *S. sclerotiorum* to promote disease will be interesting to determine in future studies. Additionally, the *A. thaliana FPA* gene was found within the LD block at Qrss2.2. *FPA* encodes an RNA-binding protein that influences flowering time but has also recently been shown to control the expression of many *A. thaliana* NB-LRR genes through premature cleavage and polyadenylation of their transcripts (Parker et al., 2021). Thus, it is tempting to speculate that *FPA* may be the gene underlying *S. sclerotiorum* resistance or susceptibility at this locus and may influence resistance through modulating the expression of NB-LRRs exploited by the pathogen to promote disease. Finally, *BAK1* (*BRASSINOSTEROID INSENSITIVE1-ASSOCIATED RECEPTOR KINASE1*), encoding a receptor-like kinase involved in pattern-triggered immunity signaling, was present in the LD block at Qrss4.4. BAK1 has previously been shown to play a role in resistance to *S. sclerotiorum*, potentially by mediating perception of the SCFE1 elicitor by RLP30 (Zhang et al., 2013a).

The availability of high-quality reference genomes for agriculturally important crop hosts along with improvements in genetic mapping promoted by high-throughput sequencing have combined to accelerate identification of loci contributing to *S. sclerotiorum* resistance in crop plants. The degree to which resistance to *S. sclerotiorum* is controlled by similar genes and/or molecular mechanisms across plant lineages representing the broad range of hosts for this pathogen remains an interesting question driving future research. Follow-up studies to identify genes underlying loci associated with resistance in this study will provide new information to help address this question. In summary, the results of this study indicated that disease evaluations with *S. sclerotiorum* isolates differing in aggressiveness identified distinct loci associated with resistance. The study additionally identified a subunit of the Mediator complex contributing to *S. sclerotiorum* resistance in *A. thaliana* and identified a large number of additional resistance-associated loci that should provide important information about resistance to this destructive pathogen in future studies.

## Data Availability

*A. thaliana* imputed SNP genotyping data (Arouisse et al., 2020) used in this study are publicly available at https://figshare.com/articles/dataset/arabidopsis_2029_Maf001_filter95/11346875/1).

## Supplementary material

Supplementary Figure 1 Plots of mean disease ratings for six *S. sclerotiorum* isolates at 4-dpi **(A)** and 7-dpi **(B)** on *A. thaliana* ecotype Col-0. Error bars indicate SEM (n = 18) and letters above bars indicate statistical groupings (generalized linear mixed model and Tukey’s *post-hoc* test, *P* < 0.05).

Supplementary Figure 2 Quantile-quantile plots of genome-wide association for *A. thaliana* resistance to *S. sclerotiorum* isolate 1980 at 4-dpi and 7-dpi determined using GEMMA **(A, B)** or FarmCPU **(C, D)**.

Supplementary Figure 3 Quantile-quantile plots of genome-wide association for *A. thaliana* resistance to *S. sclerotiorum* isolate BN325 at 4-dpi and 7-dpi determined using GEMMA **(A, B)** or FarmCPU **(C, D)**.

Supplementary Figure 4 Plots of mean disease ratings for the parent *A. thaliana* ecotype Col-0 and the *med20a* (At2g28230) mutant at 4-dpi **(A)** and 7-dpi **(B)** with *S. sclerotiorum* isolate 1980. Error bars indicate SEM (n = 45). Asterisks indicate statistically significant differences (generalized linear mixed model and Dunnett’s test, *P* < 0.01).

Supplementary Figure 5 Multiple sequence alignment of the *MED20a* promoter and coding regions for five susceptible *A. thaliana* ecotypes Col-0, Bg-2, Lm-2, Shahdara, and TOU-I-17, and four partially resistant ecotypes Ag-0, Dra-2, UKSE06-414, and Zdr-6, constructed using Clustal Omega.

Supplementary Table 2 Primers for Sanger sequencing of *MED20a* from nine *A. thaliana* ecotypes.
