# Supplementary figures and images for: Natural expression variation for the Arabidopsis MED20a mediator complex subunit influences quantitative resistance to Sclerotinia sclerotiorum
Source: Front Plant Sci. 2025 Nov 17;16:1706963. doi: 10.3389/fpls.2025.1706963 (PMC12667439; doi:10.3389/fpls.2025.1706963)

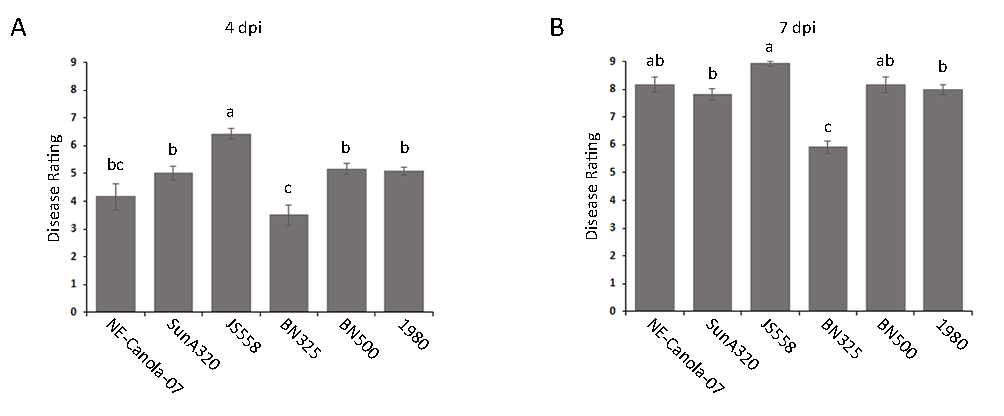

Supplement: Supplementary Figure 1 — Plots of mean disease ratings for six S. sclerotiorum isolates at 4-dpi (A) and 7-dpi (B) on A. thaliana ecotype Col-0. Error bars indicate SEM (n = 18) and letters above bars indicate statistical groupings (generalized linear mixed model and Tukey’s post-hoc test, P < 0.05). [file Image1.tif]

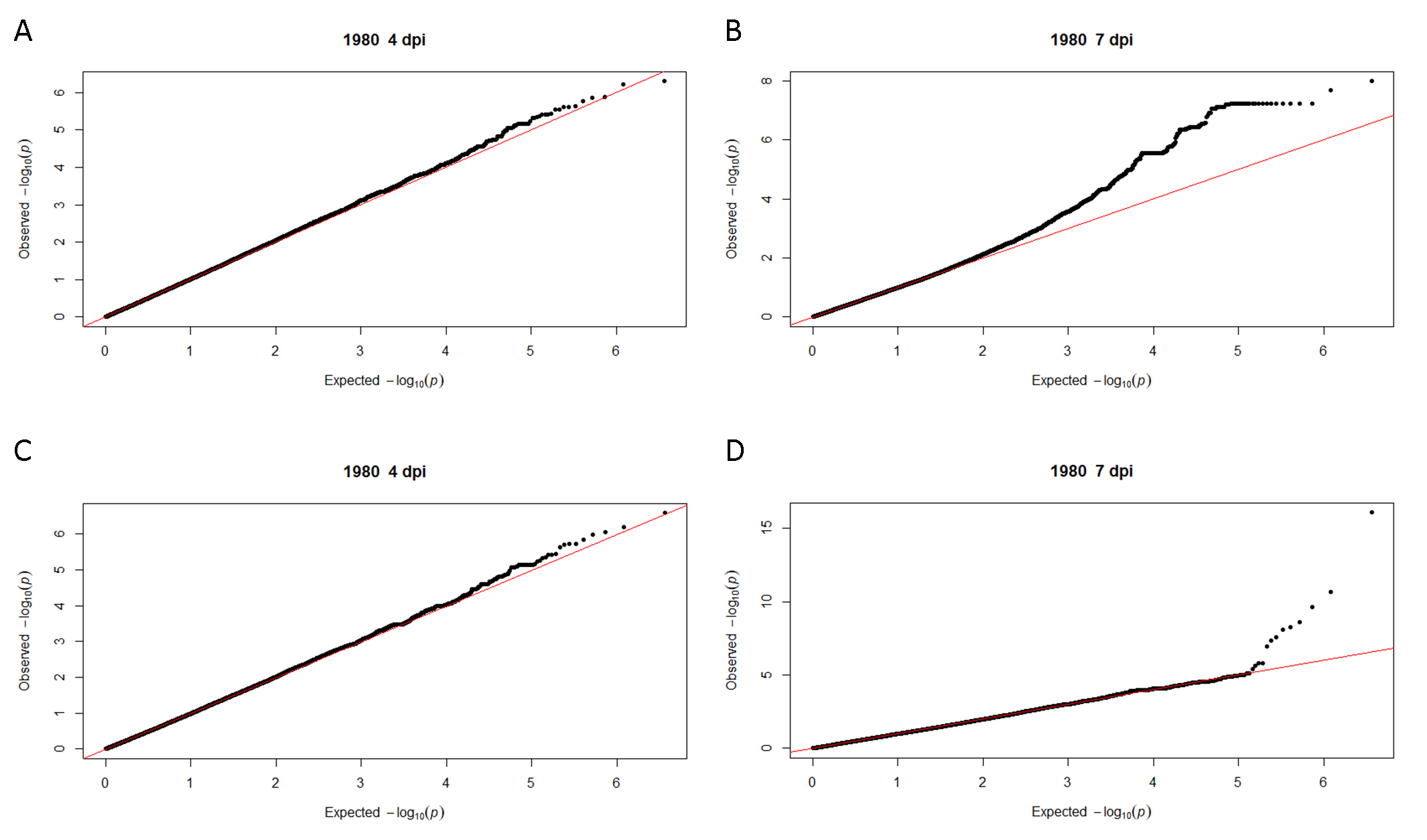

Supplement: Supplementary Figure 2 — Quantile-quantile plots of genome-wide association for A. thaliana resistance to S. sclerotiorum isolate 1980 at 4-dpi and 7-dpi determined using GEMMA (A, B) or FarmCPU (C, D). [file Image2.tif]

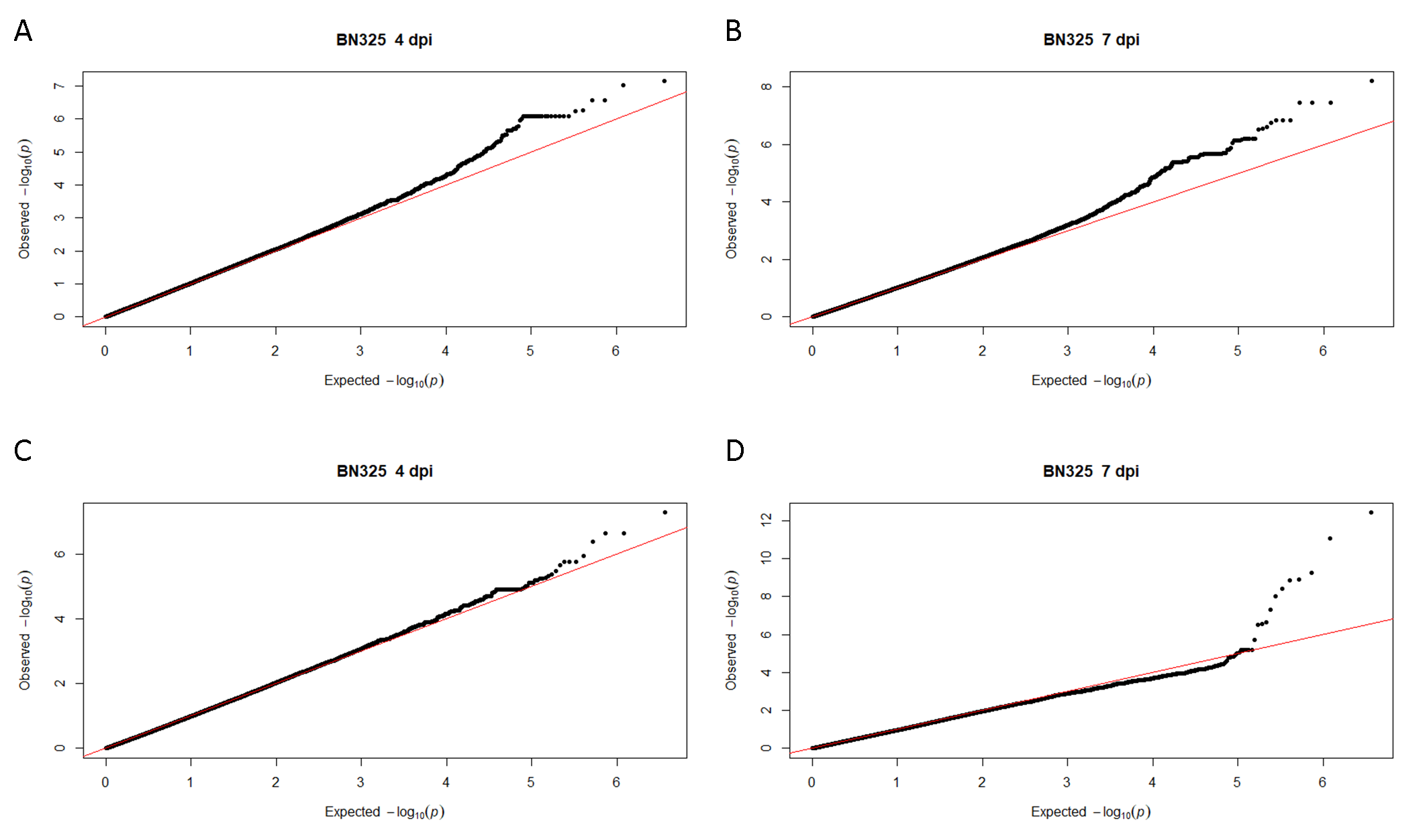

Supplement: Supplementary Figure 3 — Quantile-quantile plots of genome-wide association for A. thaliana resistance to S. sclerotiorum isolate BN325 at 4-dpi and 7-dpi determined using GEMMA (A, B) or FarmCPU (C, D). [file Image3.tif]

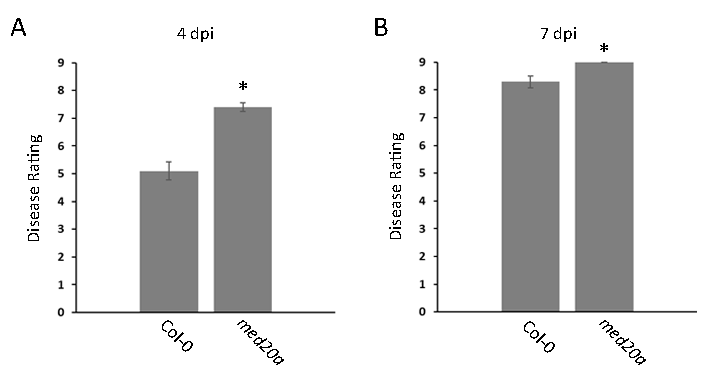

Supplement: Supplementary Figure 4 — Plots of mean disease ratings for the parent A. thaliana ecotype Col-0 and the med20a (At2g28230) mutant at 4-dpi (A) and 7-dpi (B) with S. sclerotiorum isolate 1980. Error bars indicate SEM (n = 45). Asterisks indicate statistically significant differences (generalized linear mixed model and Dunnett’s test, P < 0.01). [file Image4.tif]
