# Supplementary material for: Natural expression variation for the Arabidopsis MED20a mediator complex subunit influences quantitative resistance to Sclerotinia sclerotiorum
Source: Front Plant Sci. 2025 Nov 17;16:1706963. doi: 10.3389/fpls.2025.1706963 (PMC12667439; doi:10.3389/fpls.2025.1706963)
Supplement: Supplementary Figure 5 — Multiple sequence alignment of the MED20a promoter and coding regions for five susceptible A. thaliana ecotypes Col-0, Bg-2, Lm-2, Shahdara, and TOU-I-17, and four partially resistant ecotypes Ag-0, Dra-2, UKSE06-414, and Zdr-6, constructed using Clustal Omega. [file Image5.pdf]

```
CLUSTAL O(1.2.4) multiple sequence alignment


At2g28230|Col-0         TCCCTTGTTCTCTGCTCAGCTTCTCCAGCTCGTGGTCACATATGTCACTGAACGAAGATA  60
At2g28230|Bg-2          TCCCTTGTTCTCTGCTCAGCTTCTCCAGCTCGTGGTCACATATGTCACTGAACGAAGATA  60
At2g28230|Lm-2          TCCCTTGTTCTCTGCTCAGCTTCTCCAGCTCGTGGTCACATATGTCACTGAACGAAGATA  60
At2g28230|Shahdara      TCCCTTGTTCTCTGCTCAGCTTCTCCAGCTCGTGGTCACATATGTCACTGAACGAAGATA  60
At2g28230|TOU-I-17      TCCCTTGTTCTCTGCTCAGCTTCTCCAGCTCGTGGTCACATATGTCACTGAACGAAGATA  60
At2g28230|Ag-0          TCCCTTGTTCTCTGCTCAGCTTCTCCAGCTCGTGGTCACATATGTCACTGAACGAAGATA  60
At2g28230|Dra-2         TCCCTTGTTCTCTGCTCAGCTTCTCCAGCTCGTGGTCACATATGTCACTGAACGAAGATA  60
At2g28230|UKSE06-414    TCCCTTGTTCTCTGCTCAGCTTCTCCAGCTCGTGGTCACATATGTCACTGAACGAAGATA  60
At2g28230|Zdr-6         TCCCTTGTTCTCTGCTCAGCTTCTCCAGCTCGTGGTCACATATGTCACTGAACGAAGATA  60
                        ************************************************************

At2g28230|Col-0         AATGTAACCGAGGACTCTGTCCCATATGATTGTTGGGAAGAGACGTAACCGCTGATGCAT  120
At2g28230|Bg-2          AATGTAACCGAGGACTCTGTCCCATATGATTGTTGGGAAGAGACGTAACCGCTGATGCAT  120
At2g28230|Lm-2          AATGTAACCGAGGACTCTGTCCCATATGATTGTTGGGAAGAGACGTAACCGCTGATGCAT  120
At2g28230|Shahdara      AATGTAACCGAGGACTCTGTCCCATATGATTGTTGGGAAGAGACGTAACCGCTGATGCAT  120
At2g28230|TOU-I-17      AATGTAACCGAGGACTCTGTCCCATATGATTGTTGGGAAGAGACGTAACCGCTGATGCAT  120
At2g28230|Ag-0          AATGTAACCGAGGACTCTGTCCCATATGATTGTTGGGAAGAGACGTAACCGCTGATGCAT  120
At2g28230|Dra-2         AATGTAACCGAGGACTCTGTCCCATATGATTGTTGGGAAGAGACGTAACCGCTGATGCAT  120
At2g28230|UKSE06-414    AATGTAACCGAGGACTCTGTCCCATATGATTGTTGGGAAGAGACGTAACCGCTGATGCAT  120
At2g28230|Zdr-6         AATGTAACCGAGGACTCTGTCCCATATGATTGTTGGGAAGAGACGTAACCGCTGATGCAT  120
                        ************************************************************

At2g28230|Col-0         TGACTGTTAATCTGCTATGTAAGACGTTAGGAGGCTGAACACCACCTTCAACGACATCCT  180
At2g28230|Bg-2          TGACTGTTAATCTGCTATGTAAGACGTTAGGAGGCTGAACACCACCTTCAACGACATCCT  180
At2g28230|Lm-2          TGACTGTTAATCTGCTATGTAAGACGTTAGGAGGCTGAACACCACCTTCAACGACATCCT  180
At2g28230|Shahdara      TGACTGTTAATCTGCTATGTAAGACGTTAGGAGGCTGAACACCACCTTCAACGACATCCT  180
At2g28230|TOU-I-17      TGACTGTTAATCTGCTATGTAAGACGTTAGGAGGCTGAACACCACCTTCAACGACATCCT  180
At2g28230|Ag-0          TGACTGTTAATCTGCTATGTAAGACGTTAGGAGGCTGAACACCACCTTCAACGACATCCT  180
At2g28230|Dra-2         TGACTGTTAATCTGCTATGTAAGACGTTAGGAGGCTGAACACCACCTTCAACGACATCCT  180
At2g28230|UKSE06-414    TGACTGTTAATCTGCTATGTAAGACGTTAGGAGGCTGAACACCACCTTCAACGACATCCT  180
At2g28230|Zdr-6         TGACTGTTAATCTGCTATGTAAGACGTTAGGAGGCTGAACACCACCTTCAACGACATCCT  180
                        ************************************************************

At2g28230|Col-0         CTGAATGGTTAACTACACAACGATCACAACATACAAAAATCTTAACATTCTAGTACACAC  240
At2g28230|Bg-2          CTGAATGGTTAACTACACAACGATCACAACATACAAAAATCTTAACATTCTAGTACACAC  240
At2g28230|Lm-2          CTGAATGGTTAACTACACAACGATCACAACATACAAAAATCTTAACATTCTAGTACACAC  240
At2g28230|Shahdara      CTGAATGGTTAACTACACAACGATCACAACATACAAAAATCTTAACATTCTAGTACACAC  240
At2g28230|TOU-I-17      CTGAATGGTTAACTACACAACGATCACAACATACAAAAATCTTAACATTCTAGTACACAC  240
At2g28230|Ag-0          CTGAATGGTTAACTACACAACGATCACAACATACAAAAATCTTAACATTCTAGTACAAAC  240
At2g28230|Dra-2         CTGAATGGTTAACTACACAACGATCACAACATACAAAAATCTTAACATTCTAGTACAAAC  240
At2g28230|UKSE06-414    CTGAATGGTTAACTACACAACGATCACAACATACAAAAATCTTAACATTCTAGTACAAAC  240
At2g28230|Zdr-6         CTGAATGGTTAACTACACAACGATCACAACATACAAAAATCTTAACATTCTAGTACAAAC  240
                        ********************************************************* **

At2g28230|Col-0         CAACTATAATTAAAGCATAGCGATCACTATCAATGGGCAGATCCTGACCTAAAGGAGTCG  300
At2g28230|Bg-2          CAACTATAATTAAAGCATAGCGATCACTATCAATGGGCAGATCCTGACCTAAAGGAGTCG  300
At2g28230|Lm-2          CAACTATAATTAAAGCATAGCGATCACTATCAATGGGCAGATCCTGACCTAAAGGAGTCG  300
At2g28230|Shahdara      CAACTATAATTAAAGCATAGCGATCACTATCAATGGGCAGATCCTGACCTAAAGGAGTCG  300
At2g28230|TOU-I-17      CAACTATAATTAAAGCATAGCGATCACTATCAATGGGCAGATCCTGACCTAAAGGAGTCG  300
At2g28230|Ag-0          CAACTATAATTAAAGCATAGCGATCACTATCAATGGGCAGATCCTGACCTAAAGGAGTCG  300
At2g28230|Dra-2         CAACTATAATTAAAGCATAGCGATCACTATCAATGGGCAGATCCTGACCTAAAGGAGTCG  300
At2g28230|UKSE06-414    CAACTATAATTAAAGCATAGCGATCACTATCAATGGGCAGATCCTGACCTAAAGGAGTCG  300
At2g28230|Zdr-6         CAACTATAATTAAAGCATAGCGATCACTATCAATGGGCAGATCCTGACCTAAAGGAGTCG  300
                        ************************************************************

At2g28230|Col-0         ATGTAGAAGTCCAATGCTCCGCTGCTGTTGTGATATTTTGGACGGGAACTGACGAAGATG  360
At2g28230|Bg-2          ATGTAGAAGTCCAATGCTCCGCTGCTGTTGTGATATTTTGGACGGGAACTGACGAAGATG  360
At2g28230|Lm-2          ATGTAGAAGTCCAATGCTCCGCTGCTGTTGTGATATTTTGGACGGGAACTGACGAAGATG  360
At2g28230|Shahdara      ATGTAGAAGTCCAATGCTCCGCTGCTGTTGTGATATTTTGGACGGGAACTGACGAAGATG  360
At2g28230|TOU-I-17      ATGTAGAAGTCCAATGCTCCGCTGCTGTTGTGATATTTTGGACGGGAACTGACGAAGATG  360
At2g28230|Ag-0          ATGTAGAAGTCCAATGCTCCGCTGCTGTTGTGATATTTTGGACGGGAACTGACGAAGATG  360
At2g28230|Dra-2         ATGTAGAAGTCCAATGCTCCGCTGCTGTTGTGATATTTTGGACGGGAACTGACGAAGATG  360
At2g28230|UKSE06-414    ATGTAGAAGTCCAATGCTCCGCTGCTGTTGTGATATTTTGGACGGGAACTGACGAAGATG  360
At2g28230|Zdr-6         ATGTAGAAGTCCAATGCTCCGCTGCTGTTGTGATATTTTGGACGGGAACTGACGAAGATG  360
                        ************************************************************

At2g28230|Col-0         GTGGACAACCCGTACCTTGAGCTCTCTGTAGACGAAAAATCTAATTCGTAAGCACAATAA  420
At2g28230|Bg-2          GTGGACAACCCGTACCTTGAGCTCTCTGTAGACGAAAAATCTAATTCGTAAGCACAATAA  420
At2g28230|Lm-2          GTGGACAACCCGTACCTTGAGCTCTCTGTAGACGAAAAATCTAATTCGTAAGCACAATAA  420
At2g28230|Shahdara      GTGGACAACCCGTACCTTGAGCTCTCTGTAGACGAAAAATCTAATTCGTAAGCACAATAA  420
At2g28230|TOU-I-17      GTGGACAACCCGTACCTTGAGCTCTCTGTAGACGAAAAATCTAATTCGTAAGCACAATAA  420
At2g28230|Ag-0          GTGGACAACCCGTACCTTGAGCTCTCTGTAGACGAAAAATCTAATTCGTAAGCACAATAA  420
At2g28230|Dra-2         GTGGACAACCCGTACCTTGAGCTCTCTGTAGACGAAAAATCTAATTCGTAAGCACAATAA  420
At2g28230|UKSE06-414    GTGGACAACCCGTACCTTGAGCTCTCTGTAGACGAAAAATCTAATTCGTAAGCACAATAA  420
At2g28230|Zdr-6         GTGGACAACCCGTACCTTGAGCTCTCTGTAGACGAAAAATCTAATTCGTAAGCACAATAA  420
                        ************************************************************
```

```
At2g28230|Col-0           ACGATCTGAAACCAACAAAGAGTAACAGTCGTTTTCAAGAAGCAGCAAGTAACCTGATCT  480
At2g28230|Bg-2            ACGATCTGAAACCAACAAAGAGTAACAGTCGTTTTCAAGAAGCAGCAAGTAACCTGATCT  480
At2g28230|Lm-2            ACGATCTGAAACCAACAAAGAGTAACAGTCGTTTTCAAGAAGCAGCAAGTAACCTGATCT  480
At2g28230|Shahdara        ACGATCTGAAACCAACAAAGAGTAACAGTCGTTTTCAAGAAGCAGCAAGTAACCTGATCT  480
At2g28230|TOU-I-17        ACGATCTGAAACCAACAAAGAGTAACAGTCGTTTTCAAGAAGCAGCAAGTAACCTGATCT  480
At2g28230|Ag-0            ACGATCTGAAACCAACAAAGAGTAACAGTCGTTTTCAAGAAGCAGCAAGTAACCTGATCT  480
At2g28230|Dra-2           ACGATCTGAAACCAACAAAGAGTAACAGTCGTTTTCAAGAAGCAGCAAGTAACCTGATCT  480
At2g28230|UKSE06-414      ACGATCTGAAACCAACAAAGAGTAACAGTCGTTTTCAAGAAGCAGCAAGTAACCTGATCT  480
At2g28230|Zdr-6           ACGATCTGAAACCAACAAAGAGTAACAGTCGTTTTCAAGAAGCAGCAAGTAACCTGATCT  480
                          ************************************************************

At2g28230|Col-0           GATCTAGCAGAACCTTGAATATCTAATATAGAGGCATGGTCTGGAAAACGACTCGAAAAC  540
At2g28230|Bg-2            GATCTAGCAGAACCTTGAATATCTAATATAGAGGCATGGTCTGGAAAACGNNNNNNNNNN  540
At2g28230|Lm-2            GATCTAGCAGAACCTTGAATATCTAATATAGAGGCATGGTCTGGAAAACG----------  530
At2g28230|Shahdara        GATCTAGCAGAACCTTGAATATCTAATATAGAGGCATGGTCTGGAAAACG----------  530
At2g28230|TOU-I-17        GATCTAGCAGAACCTTGAATATCTAATATAGAGGCATGGTCTGGAAAACGACTCGAAAAC  540
At2g28230|Ag-0            GATCTAGCAGAACCTTGAATATCTAATATAGAGGCATGGTCTGGAAAACGACTCGAAAAC  540
At2g28230|Dra-2           GATCTAGCAGAACCTTGAATATCTAATATAGAGGCATGGTCTGGAAAACGACTCGAAAAC  540
At2g28230|UKSE06-414      GATCTAGCAGAACCTTGAATATCTAATATAGAGGCATGGTCTGGAAAACG----------  530
At2g28230|Zdr-6           GATCTAGCAGAACCTTGAATATCTAATATAGAGGCATGGTCTGGAAAAC-----------  529
                          *************************************************

At2g28230|Col-0           TCTTGAACCAAAATGGCATCAGCATCATGACTTGTTCTATTACTTTCTTCTCTAATCAAC  600
At2g28230|Bg-2            N-----NNNNNNNNGGCATCAGCATCATGACTTGTTCTATTACTTTCTTCTCTAATCAAC  595
At2g28230|Lm-2            -----------AATGGCATCAGCATCATGACTTGTTCTATTACTTTCTTCTCTAATCAAC  579
At2g28230|Shahdara        -----------AATGGCATCAGCATCATGACTTGTTCTATTACTTTCTTCTCTAATCAAC  579
At2g28230|TOU-I-17        TCTTGAACCAAAATGGCATCAGCATCATGACTTGTTCTATTACTTTCTTCTCTAATCAAC  600
At2g28230|Ag-0            TCTTGAACCAAAATGGCATCAGCATCATGACTTGTTCTATTACTTTCTTCTCTAATCAAC  600
At2g28230|Dra-2           TCTTGAACCAAAATGGCATCAGCATCATGACTTGTTCTATTACTTTCTTCTCTAATCAAC  600
At2g28230|UKSE06-414      -----------AATGGCATCAGCATCATGACTTGTTCTATTACTTTCTTCTCTAATCAAC  579
At2g28230|Zdr-6           -----------GATGGCATCAGCATCATGACTTGTTCTATTACTTTCTTCTCTAATCAAC  578
                                        **********************************************

At2g28230|Col-0           GGCGCTTCCTCTACATTCAAAGGTAACTCTAAGGAAGCAGCTCCGGAAACTGAAACCGGT  660
At2g28230|Bg-2            GGCGCTTCCTCTACATTCAAAGGTAACTCTAAGGAAGCAGCTCCGGAAACTGAAACCGGT  655
At2g28230|Lm-2            GGCGCTTCCTCTACATTCAAAGGTAACTCTAAGGAAGCAGCTCCGGAAACTGAAACCGGT  639
At2g28230|Shahdara        GGCGCTTCCTCTACATTCAAAGGTAACTCTAAGGAAGCAGCTCCGGAAACTGAAACCGGT  639
At2g28230|TOU-I-17        GGCGCTTCCTCTACATTCAAAGGTAACTCTAAGGAAGCAGCTCCGGAAACTGAAACCGGT  660
At2g28230|Ag-0            GGCGCTTCCTCTACATTCAAAGGTAACTCTAAGGAAGCAGCTCCGGAAACTGAAACCGGT  660
At2g28230|Dra-2           GGCGCTTCCTCTACATTCAAAGGTAACTCTAAGGAAGCAGCTCCGGAAACTGAAACCGGT  660
At2g28230|UKSE06-414      GGCGCTTCCTCTACATTCAAAGGTAACTCTAAGGAAGCAGCTCCGGAAACTGAAACCGGT  639
At2g28230|Zdr-6           GGCGCTTCCTCTACATTCAAAGGTAACTCTAAGGAAGCAGCTCCGGAAACTGAAACCGGT  638
                          ************************************************************

At2g28230|Col-0           GGCATCACTGTGTCACAAACCATATCTTCAGTCAAAACCTCAGGTATATTTGCTGAAGAC  720
At2g28230|Bg-2            GGCATCACTGTGTCACAAACCATATCTTCAGTCAAAACCTCAGGTATATTTGCTGAAGAC  715
At2g28230|Lm-2            GGCATCACTGTGTCACAAACCATATCTTCAGTCAAAACCTCAGGTATATTTGCTGAAGAC  699
At2g28230|Shahdara        GGCATCACTGTGTCACAAACCATATCTTCAGTCAAAACCTCAGGTATATTTGCTGAAGAC  699
At2g28230|TOU-I-17        GGCATCACTGTGTCACAAACCATATCTTCAGTCAAAACCTCAGGTATATTTGCTGAAGAC  720
At2g28230|Ag-0            GGCATCACTGTGTCACAAACCATATCTTCAGTCAAAACCTCAGGTATATTTGCTGAAGAC  720
At2g28230|Dra-2           GGCATCACTGTGTCACAAACCATATCTTCAGTCAAAACCTCAGGTATATTTGCTGAAGAC  720
At2g28230|UKSE06-414      GGCATCACTGTGTCACAAACCATATCTTCAGTCAAAACCTCAGGTATATTTGCTGAAGAC  699
At2g28230|Zdr-6           GGCATCACTGTGTCACAAACCATATCTTCAGTCAAAACCTCAGGTATATTTGCTGAAGAC  698
                          ************************************************************

At2g28230|Col-0           ACCATTCCAGGCCCATCTAGTTGAGTACTACCCGATTTACTCTGCGCTTTTGGTAAAGAA  780
At2g28230|Bg-2            ACCATTCCAGGCCCATCTAGTTGAGTACTACCCGATTTACTCTGCGCTTTTGGTAAAGAA  775
At2g28230|Lm-2            ACCATTCCAGGCCCATCTAGTTGAGTACTACCCGATTTACTCTGCGCTTTTGGTAAAGAA  759
At2g28230|Shahdara        ACCATTCCAGGCCCATCTAGTTGAGTACTACCCGATTTACTCTGCGCTTTTGGTAAAGAA  759
At2g28230|TOU-I-17        ACCATTCCAGGCCCATCTAGTTGAGTACTACCCGATTTACTCTGCGCTTTTGGTAAAGAA  780
At2g28230|Ag-0            ACCATTCCAGGCCCATCTAGTTGAGTACTACCCGATTTACTCTGCGCTTTTGGTAAAGAA  780
At2g28230|Dra-2           ACCATTCCAGGCCCATCTAGTTGAGTACTACCCGATTTACTCTGCGCTTTTGGTAAAGAA  780
At2g28230|UKSE06-414      ACCATTCCAGGCCCATCTAGTTGAGTACTACCCGATTTACTCTGCGCTTTTGGTAAAGAA  759
At2g28230|Zdr-6           ACCATTCCAGGCCCATCTAGTTGAGTACTACCCGATTTACTCTGCGCTTTTGGTAAAGAA  758
                          ************************************************************

At2g28230|Col-0           TCTGTAACTTCTTGAGTTTCCATTGGAACCATAACATCAAATTGCTTTCACCAGTGAAGA  840
At2g28230|Bg-2            TCTGTAACTTCTTGAGTTTCCATTGGAACCATAACATCAAATTGCTTTCACCAGTGAAGA  835
At2g28230|Lm-2            TCTGTAACTTCTTGAGTTTCCATTGGAACCATAACATCAAATTGCTTTCACCAGTGAAGA  819
At2g28230|Shahdara        TCTGTAACTTCTTGAGTTTCCATTGGAACCATAACATCAAATTGCTTTCACCAGTGAAGA  819
At2g28230|TOU-I-17        TCTGTAACTTCTTGAGTTTCCATTGGAACCATAACATCAAATTGCTTTCACCAGTGAAGA  840
At2g28230|Ag-0            TCTGTAACTTCTTGAGTTTCCATTGGAACCATAACATCAAATTGCTTTCACCAGTGAAGA  840
At2g28230|Dra-2           TCTGTAACTTCTTGAGTTTCCATTGGAACCATAACATCAAATTGCTTTCACCAGTGAAGA  840
At2g28230|UKSE06-414      TCTGTAACTTCTTGAGTTTCCATTGGAACCATAACATCAAATTGCTTTCACCAGTGAAGA  819
At2g28230|Zdr-6           TCTGTAACTTCTTGAGTTTCCATTGGAACCATAACATCAAATTGCTTTCACCAGTGAAGA  818
                          ************************************************************

At2g28230|Col-0           GGTTTAGCTACACATCATAACATATCTCAGTCTATTAGAGAAAACAAAACCAAGCTATTC  900
At2g28230|Bg-2            GGTTTAGCTACACATCATAACATATCTCAGTCTATTAGAGAAAACAAAACCAAGCTATTC  895
At2g28230|Lm-2            GGTTTAGCTACACATCATAACATATCTCAGTCTATTAGAGAAAACAAAACCAAGCTATTC  879
At2g28230|Shahdara        GGTTTAGCTACACATCATAACATATCTCAGTCTATTAGAGAAAACAAAACCAAGCTATTC  879
```

```
At2g28230|TOU-I-17        GGTTTAGCTACACATCATAACATATCTCAGTCTATTAGAGAAAACAAAACCAAGCTATTC  900
At2g28230|Ag-0            GGTTTAGCTACACATCATAACATATCTCAGTCTATTAGAGAAAACAAAACCAAGCTATTC  900
At2g28230|Dra-2           GGTTTAGCTACACATCATAACATATCTCAGTCTATTAGAGAAAACAAAACCAAGCTATTC  900
At2g28230|UKSE06-414      GGTTTAGCTACACATCATAACATATCTCAGTCTATTAGAGAAAACAAAACCAAGCTATTC  879
At2g28230|Zdr-6           GGTTTAGCTACACATCATAACATATCTCAGTCTATTAGAGAAAACAAAACCAAGCTATTC  878
                          ************************************************************

At2g28230|Col-0           AAATTTACAATCTTTGAAGAACACAAACACACTAATCACCGAGAAGTTGAAAATGAGAGT  960
At2g28230|Bg-2            AAATTTACAATCTTTGAAGAACACAAACACACTAATCACCGAGAAGTTGAAAATGAGAGT  955
At2g28230|Lm-2            AAATTTACAATCTTTGAAGAACACAAACACACTAATCACCGAGAAGTTGAAAATGAGAGT  939
At2g28230|Shahdara        AAATTTACAATCTTTGAAGAACACAAACACACTAATCACCGAGAAGTTGAAAATGAGAGT  939
At2g28230|TOU-I-17        AAATTTACAATCTTTGAAGAACACAAACACACTAATCACCGAGAAGTTGAAAATGAGAGT  960
At2g28230|Ag-0            AAATTTACAATCTTTGAAGAACACAAACACACTAATCACCGAGAAGTTGAAAATGAGAGT  960
At2g28230|Dra-2           AAATTTACAATCTTTGAAGAACACAAACACACTAATCACCGAGAAGTTGAAAATGAGAGT  960
At2g28230|UKSE06-414      AAATTTACAATCTTTGAAGAACACAAACACACTAATCACCGAGAAGTTGAAAATGAGAGT  939
At2g28230|Zdr-6           AAATTTACAATCTTTGAAGAACACAAACACACTAATCACCGAGAAGTTGAAAATGAGAGT  938
                          ************************************************************

At2g28230|Col-0           GACACTTTAACAATCATAGTAGCAAAACATAGAATCAACGAAATTAAAACCATATTATAT  1020
At2g28230|Bg-2            GACACTTTAACAATCATAGTAGCAAAACATAGAATCAACGAAATTAAAACCATATTATAT  1015
At2g28230|Lm-2            GACACTTTAACAATCATAGTAGCAAAACATAGAATCAACGAAATTAAAACCATATTATAT  999
At2g28230|Shahdara        GACACTTTAACAATCATAGTAGCAAAACATAGAATCAACGAAATTAAAACCATATTATAT  999
At2g28230|TOU-I-17        GACACTTTAACAATCATAGTAGCAAAACATAGAATCAACGAAATTAAAACCATATTATAT  1020
At2g28230|Ag-0            GACACTTTAACAATCATAGTAGCAAAACATAGAATCAACGAAATTAAAACCATATTATAT  1020
At2g28230|Dra-2           GACACTTTAACAATCATAGTAGCAAAACATAGAATCAACGAAATTAAAACCATATTATAT  1020
At2g28230|UKSE06-414      GACACTTTAACAATCATAGTAGCAAAACATAGAATCAACGAAATTAAAACCATATTATAT  999
At2g28230|Zdr-6           GACACTTTAACAATCATAGTAGCAAAACATAGAATCAACGAAATTAAAACCATATTATAT  998
                          ************************************************************

At2g28230|Col-0           ATAAAAATCGTAACTTTATTCCCAAATTTCATATATGCTAGATTGTGAAGAACCATTTTT  1080
At2g28230|Bg-2            ATAAAAATCGTAACTTTATTCCCAAATTTCATATATGCTAGATTGTGAAGAACCATTTTT  1075
At2g28230|Lm-2            ATAAAAATCGTAACTTTATTCCCAAATTTCATATATGCTAGATTGTGAAGAACCATTTTT  1059
At2g28230|Shahdara        ATAAAAATCGTAACTTTATTCCCAAATTTCATATATGCTAGATTGTGAAGAACCATTTTT  1059
At2g28230|TOU-I-17        ATAAAAATCGTAACTTTATTCCCAAATTTCATATATGCTAGATTGTGAAGAACCATTTTT  1080
At2g28230|Ag-0            ATAAAAATCGTAACTTTATTCCCAAATTTCATATATGCTAGATTGTGAAGAACCATTTTT  1080
At2g28230|Dra-2           ATAAAAATCGTAACTTTATTCCCAAATTTCATATATGCTAGATTGTGAAGAACCATTTTT  1080
At2g28230|UKSE06-414      ATAAAAATCGTAACTTTATTCCCAAATTTCATATATGCTAGATTGTGAAGAACCATTTTT  1059
At2g28230|Zdr-6           ATAAAAATCGTAACTTTATTCCCAAATTTCATATATGCTAGATTGTGAAGAACCATTTTT  1058
                          ************************************************************

At2g28230|Col-0           TTCAATTCGATTTTGAAATAAAAAAGATCCGTGAAAATAAAATTGCGAGTTATTGTTTGA  1140
At2g28230|Bg-2            TTCAATTCGATTTTGAAATAAAAAAGATCCGTGAAAATAAAATTGCGAGTTATTGTTTGA  1135
At2g28230|Lm-2            TTCAATTCGATTTTGAAATAAAAAAGATCCGTGAAAATAAAATTGCGAGTTATTGTTTGA  1119
At2g28230|Shahdara        TTCAATTCGATTTTGAAATAAAAAAGATCCGTGAAAATAAAATTGCGAGTTATTGTTTGA  1119
At2g28230|TOU-I-17        TTCAATTCGATTTTGAAATAAAAAAGATCCGTGAAAATAAAATTGCGAGTTATTGTTTGA  1140
At2g28230|Ag-0            TTCAATTCGATTTTGAAATAAAAAAGATCCGTGAAAATAAAATTGCGAGTTATTGTTTGA  1140
At2g28230|Dra-2           TTCAATTCGATTTTGAAATAAAAAAGATCCGTGAAAATAAAATTGCGAGTTATTGTTTGA  1140
At2g28230|UKSE06-414      TTCAATTCGATTTTGA-ATAAAAAAGATCCGTGAAAATAAAATTGCGAGTTATTGTTTGA  1118
At2g28230|Zdr-6           TTCAATTCGATTTTGAAATAAAAAAGATCCGTGAAAATAAAATTGCGAGTTATTGTTTGA  1118
                          **************** *******************************************

At2g28230|Col-0           GAATTGAAAGAGAAGAAAACCCAGAACCGAAAATAGACTTAACCCTACCTTAAAATGAAG  1200
At2g28230|Bg-2            GAATTGAAAGAGAAGAAAACCCAGACCCGAAAATAGACTTAACCCTACCTTAAAATGAAG  1195
At2g28230|Lm-2            GAATTGAAAGAGAAGAAAACCCAGACCCGAAAATAGACTTAACCCTACCTTAAAATGAAG  1179
At2g28230|Shahdara        GAATTGAAAGAGAAGAAAACCCAGAACCGAAAATAGACTTAACCCTACCTTAAAATGAAG  1179
At2g28230|TOU-I-17        GAATTGAAAGAGAAGAAAACCCAGAACCGAAAATAGACTTAACCCTACCTTAAAATGAAG  1200
At2g28230|Ag-0            GAATTGAAAGAGAAGAAAACCCAGAACCGAAAATAGACTTAACCCTACCTTAAAATGAAG  1200
At2g28230|Dra-2           GAATTGAAAGAGAAGAGAACCCAGAACCGAAAATAGACTTAACCCTACCTTAAAATGAAG  1200
At2g28230|UKSE06-414      GAATTGAAAGAGAAGAAAACCCAGAACCGAAAATAGACTTAACCCTACCTTAAAATGAAG  1178
At2g28230|Zdr-6           GAATTGAAAGAGAAGAAAACCCAGAACCGAAAATAGACTTAACCCTACCTTAAAATGAAG  1178
                          **************** ******** **********************************

At2g28230|Col-0           AAACAAATGTCGGATTCAAGGTTCTTCTTCCTCGTCTACGTGAAGTCCAGGTTAAGAACA  1260
At2g28230|Bg-2            AAACAAATGTCGGATTCAAGGTTCTTCTTCCTCGTCTACGTGAAGTCCAGGTTAAGAACA  1255
At2g28230|Lm-2            AAACAAATGTCGGATTCAAGGTTCTTCTTCCTCGTCTACGTGAAGTCCAGGTTAAGAACA  1239
At2g28230|Shahdara        AAACAAATGTCGGATTCAAGGTTCTTCTTCCTCGTCTACGTGAAGTCCAGGTTAAGAACA  1239
At2g28230|TOU-I-17        AAACAAATGTCGGATTCAAGGTTCTTCTTCCTCGTCTACGTGAAGTCCAGGTTAAGAACA  1260
At2g28230|Ag-0            AAACAAATGTCGGATTCAAGGTTCTTCTTCCTCGTCTACGTGAAGTCCAGGTTAAGAACA  1260
At2g28230|Dra-2           AAACAAATGTCGGATTCAAGGTTCTTCTTCCTCGTCTACGTGAAGTCCAGGTTAAGAACA  1260
At2g28230|UKSE06-414      AAACAAATGTCGGATTCAAGGTTCTTCTTCCTCGTCTACGTGAAGTCCAGGTTAAGAACA  1238
At2g28230|Zdr-6           AAACAAATGTCGGATTCAAGGTTCTTCTTCCTCGTCTACGTGAAGTCCAGGTTAAGAACA  1238
                          ************************************************************

At2g28230|Col-0           GTTAATAGAATCAAGAGACATGGAGTATTTAATTAAGCTGTGTTGGGCCACATTTATTAA  1320
At2g28230|Bg-2            GTTAATAGAATCAAGAGACATGGAGTATTTAATTAAGCTGTATTGGGCCACATTTATTAA  1315
At2g28230|Lm-2            GTTAATAGAATCAAGAGACATGGAGTATTTAATTAAGCTGTATTGGGCCACATTTATTAA  1299
At2g28230|Shahdara        GTTAATAGAATCAAGAGACATGGAGTATTTAATTAAGCTGTGTTGGGCCACATTTATTAA  1299
At2g28230|TOU-I-17        GTTAATAGAATCAAGAGACATGGAGTATTTAATTAAGCTGTGTTGGGCCACATTTATTAA  1320
At2g28230|Ag-0            GTTAATAGAATCAAGAGACATGGAGTATTTAATTAAGCTGTGTTGGGCCACATTTATTAA  1320
At2g28230|Dra-2           GTTAATAGAATCAAGAGACATGGAGTATTTAATTAAGCTGTGTTGGGCCACATTTATTAA  1320
At2g28230|UKSE06-414      GTTAATAGAATCAAGAGACATGGAGTATTTAATTAAGCTGTGTTGGGCCACATTTATTAA  1298
```

```
At2g28230|Zdr-6          GTTAATAGAATCAAGAGACATGGAGTATTTAATTAAGCTGTGTTGGGCCACATTTATTAA  1298
                         ***************************************** ******************

At2g28230|Col-0          GCTGTTTTGGGCCTTCGGCCCATACCTCAATTTCGATTGGGTTTGTTGATATAACATATA  1380
At2g28230|Bg-2           GCTGTTTTGGGCCTTCGGCCCCTTCCTCAATTTCGATNGGGTTTGTTGATATAACATATA  1375
At2g28230|Lm-2           GCTGTGTTGGGCCTTCGGCCCATACCTCAATTTCGATTGGGTTTGTTGATATAACATATA  1359
At2g28230|Shahdara       GCTGTGTTGGGCCTTCGGCCCATACCTCAATTTCGATTGGGTTTGTTGATATAACATATA  1359
At2g28230|TOU-I-17       GCTGTGTTGGGCCTTCGGCCCATACCTCAATTTCGATTGGGTTTGTTGATATAACATATA  1380
At2g28230|Ag-0           GCTGTGTTGGGCCTTCGGCCCATACCTCAATTTCGATTGGGTTTGTTGATATAACATATA  1380
At2g28230|Dra-2          GCTGTGTTGGGCCTTCGGCCCATACCTCAATTTCGATTGGGTTTGTTGATATAACATATA  1380
At2g28230|UKSE06-414     GCTGTGTTGGGCCTTCGGCCCATACCTCAATTTCGATTGGGTTTGTTGATATAACATATA  1358
At2g28230|Zdr-6          GCTGTGTTGGGCCTTCGGCCCATACCTCAATTTCGATTGGGTTTGTTGATATAACATATA  1358
                         ***** *************** * ************* **********************

At2g28230|Col-0          CATATGTTTTTTTATTTTTTGTTGTGTCAGTTTCGAACAGTGTTTGTTTTGTCATTCTC  1440
At2g28230|Bg-2           CATATGTTTTTTTATTTTTTGTTGTGTCAGTTTCGAACAGTGTTTGTTTTGTCATTCTC  1435
At2g28230|Lm-2           CATATGTTTTTTTATTTTTTGTTGTGTCAGTTTCGAACAGTGTTTGTTTTGTCATTCTC  1419
At2g28230|Shahdara       CATATGTTTTTTTATTTTTTGTTGTGTCAGTTTCGAACAGTGTTTGTTTTGTCATTCTC  1419
At2g28230|TOU-I-17       CATATGTTTTTTTATTTTTTGTTGTGTCAGTTTCGAACAGTGTTTGTTTTGTCATTCTC  1440
At2g28230|Ag-0           CATATGTTTTTTTATTTTTTGTTGTGTCAGTTTCGAACAGTGTTTGTTTTGTCATTCTC  1440
At2g28230|Dra-2          CATATGTTTTTTTATTTTTTGTTGTGTCAGTTTCGAACAGTGTTTGTTTTGTCATTCTC  1440
At2g28230|UKSE06-414     CATATGTTTTTTTATTTTTTGTTGTGTCAGTTTCGAACAGTGTTTGTTTTGTCATTCTC  1418
At2g28230|Zdr-6          CATATGTTTTTTTATTTTTTGTTGTGTCAGTTTCGAACAGTGTTTGTTTTGTCATTCTC  1418
                         ************************************************************

At2g28230|Col-0          CCTCAACAACAATGCCCGTCAAGTGGTAAGCTTTGATCCAATTTCGTTTTCCAAAATCTC  1500
At2g28230|Bg-2           CCTCAACAACAATGCCCGTCAAGTGGTAAGCTTTGATCCAATTTCGTTTTCCAAAATCTC  1495
At2g28230|Lm-2           CCTCAACAACAATGCCCGTCAAGTGGTAAGCTTTGATCCAATTTCGTTTTCCAAAATCTC  1479
At2g28230|Shahdara       CCTCAACAACAATGCCCGTCAAGTGGTAAGCTTTGATCCAATTTCGTTTTCCAAAATCTC  1479
At2g28230|TOU-I-17       CCTCAACAACAATGCCCGTCAAGTGGTAAGCTTTGATCCAATTTCGTTTTCCAAAATCTC  1500
At2g28230|Ag-0           CCTCAACAACAATGCCCGTCAAGTGGTAAGCTTTGATCCAATTTCGTTTTCCAAAATCTC  1500
At2g28230|Dra-2          CCTCAACAACAATGCCCGTCAAGTGGTAAGCTTTGATCCAATTTCGTTTTCCAAAATCTC  1500
At2g28230|UKSE06-414     CCTCAACAACAATGCCCGTCAAGTGGTAAGCTTTGATCCAATTTCGTTTTCCAAAATCTC  1478
At2g28230|Zdr-6          CCTCAACAACAATGCCCGTCAAGTGGTAAGCTTTGATCCAATTTCGTTTTCCAAAATCTC  1478
                         ************************************************************

At2g28230|Col-0          GATTTTTTTCGTTTCTGTTCTGATTACTCTTCCGATTCTTGTATTTGGGTTTTGTT----  1556
At2g28230|Bg-2           GATTTTTTTCGTTTCTGTTCTGATTACTCTTCCGATTCTTGTATTTGGGTTTTGTT----  1551
At2g28230|Lm-2           GATTTTTTTCGTTTCTGTTCTGATTACTCTTCCGATTCTTGTATTTGGGTTTTGTT----  1535
At2g28230|Shahdara       GATTTTTTTCGTTTCTGTTCTGATTACTCTTCCGATTCTTGTATTTGGGTTTTGTT----  1535
At2g28230|TOU-I-17       GATTTTTTTCGTTTCTGTTCTGATTACTCTTCCGATTCTTGTATTTGGGTTTTGTT----  1556
At2g28230|Ag-0           GATTTTTTTCGTTTCTGTTCTGATTACTCTTCCGATTCTTGTATTTGGGTTTTGTT----  1556
At2g28230|Dra-2          GATTTTTTTCGTTTCTGTTCTGATTACTCTTCCGATTCTTGTATGTGGGTTTTGTTTTTT  1560
At2g28230|UKSE06-414     GATTTTTTTCGTTTCTGTTCTGATTACTCTTCCGATTCTTGTATTTGGGTTTTGTT----  1534
At2g28230|Zdr-6          GATTTTTTTCGTTTCTGTTCTGATTACTCTTCCGATTCTTGTATTTGGGTTTTGTT----  1534
                         ******************************************** ***********

At2g28230|Col-0          --------TTTTTCTAGGGTTTTACATTGGCAACCAAACCAAGGATCAACAGTAAGCAGC  1608
At2g28230|Bg-2           --------TTTTTCTAGGGTTTTACATTGGCAACCAAACCAAGGATCAACAGTAAGCAGC  1603
At2g28230|Lm-2           --------TTTTTCTAGGGTTTTACATTGGCAACCAAACCAAGGATCAACAGTAAGCAGC  1587
At2g28230|Shahdara       --------TTTTTCTAGGGTTTTACATTGGCAACCAAACCAAGGATCAACAGTAAGCAGC  1587
At2g28230|TOU-I-17       --------TTTTTCTAGGGTTTTACATTGGCAACCAAACCAAGGATCAACAGTAAGCAGC  1608
At2g28230|Ag-0           --------TTTTTCTAGGGTTTTACATTGGCAACCAAACCAAGGATCAACAGTAAGCAGC  1608
At2g28230|Dra-2          GGTTTTGTTTTTTCTAGGGTTTTACATTGGCAACCAAACCAAGGATCAACAGTAAGCAGC  1620
At2g28230|UKSE06-414     --------TTTTTCTAGGGTTTTACATTGGCAACCAAACCAAGGATCAACAGTAAGCAGC  1586
At2g28230|Zdr-6          --------TTTTTCTAGGGTTTTACATTGGCAACCAAACCAAGGATCAACAGTAAGCAGC  1586
                                 ****************************************************

At2g28230|Col-0          CAAATCCTAAACGAAGCAACACAATGTGTAGAGAGCATCAATGGTGTGAAAGAAGGAAGA  1668
At2g28230|Bg-2           CAAATCCTAAACGAAGCAACACAATGTGTAGAGAGCATCAATGGTGTGAAAGAAGGAAGA  1663
At2g28230|Lm-2           CAAATCCTAAACGAAGCAACACAATGTGTAGAGAGCATCAATGGTGTGAAAGAAGGAAGA  1647
At2g28230|Shahdara       CAAATCCTAAACGAAGCAACACAATGTGTAGAGAGCATCAATGGTGTGAAAGAAGGAAGA  1647
At2g28230|TOU-I-17       CAAATCCTAAACGAAGCAACACAATGTGTAGAGAGCATCAATGGTGTGAAAGAAGGAAGA  1668
At2g28230|Ag-0           CAAATCCTAAACGAAGCAACACAATGTGTAGAGAGCATCAATGGTGTGAAAGAAGGAAGA  1668
At2g28230|Dra-2          CAAATCCTAAACGAAGCAACACAATGTGTAGAGAGCATCAATGGTGTGAAAGAAGGAAGA  1680
At2g28230|UKSE06-414     CAAATCCTAAACGAAGCAACACAATGTGTAGAGAGCATCAATGGTGTGAAAGAAGGAAGA  1646
At2g28230|Zdr-6          CAAATCCTAAACGAAGCAACACAATGTGTAGAGAGCATCAATGGTGTGAAAGAAGGAAGA  1646
                         ************************************************************

At2g28230|Col-0          TGGAAAGCAACACTCAACTATTACAAACCCATGTTAAAAGACCAAGCGAATCAATTGGAG  1728
At2g28230|Bg-2           TGGAAAGCAACACTCAACTATTACAAACCCATGTTAAAAGACCAAGCGAATCAATTGGAG  1723
At2g28230|Lm-2           TGGAAAGCAACACTCAACTATTACAAACCCATGTTAAAAGACCAAGCGAATCAATTGGAG  1707
At2g28230|Shahdara       TGGAAAGCAACACTCAACTATTACAAACCCATGTTAAAAGACCAAGCGAATCAATTGGAG  1707
At2g28230|TOU-I-17       TGGAAAGCAACACTCAACTATTACAAACCCATGTTAAAAGACCAAGCGAATCAATTGGAG  1728
At2g28230|Ag-0           TGGAAAGCAACACTCAACTATTACAAACCCATGTTAAAAGACCAAGCGAATCAATTGGAG  1728
At2g28230|Dra-2          TGGAAAGCAACACTCAACTATTACAAACCCATGTTAAAAGACCAAGCGAATCAATTGGAG  1740
At2g28230|UKSE06-414     TGGAAAGCAACACTCAACTATTACAAACCCATGTTAAAAGACCAAGCGAATCAATTGGAG  1706
At2g28230|Zdr-6          TGGAAAGCAACACTCAACTATTACAAACCCATGTTAAAAGACCAAGCGAATCAATTGGAG  1706
                         ************************************************************

At2g28230|Col-0          TTTCCTCGTGATTTTCTAGGGATTTCACTTGCGGATCAGCCGAATAAGTACTATTTCATT  1788
```

```
At2g28230|Bg-2          TTTCCTCGTGATTTTCTAGGGATTTCACTTGCGGATCAGCCGAATAAGTACTATTTCATT  1783
At2g28230|Lm-2          TTTCCTCGTGATTTTCTAGGGATTTCACTTGCGGATCAGCCGAATAAGTACTATTTCATT  1767
At2g28230|Shahdara      TTTCCTCGTGATTTTCTAGGGATTTCACTTGCGGATCAGCCGAATAAGTACTATTTCATT  1767
At2g28230|TOU-I-17      TTTCCTCGTGATTTTCTAGGGATTTCACTTGCGGATCAGCCGAATAAGTACTATTTCATT  1788
At2g28230|Ag-0          TTTCCTCGTGATTTTCTAGGGATTTCACTTGCGGATCAGCCGAATAAGTACTATTTCATT  1788
At2g28230|Dra-2         TTTCCTCGTGATTTTCTAGGGATTTCACTTGCGGATCAGCCGAATAAGTACTATTTCATT  1800
At2g28230|UKSE06-414    TTTCCTCGTGATTTTCTAGGGATTTCACTTGCGGATCAGCCGAATAAGTACTATTTCATT  1766
At2g28230|Zdr-6         TTTCCTCGTGATTTTCTAGGGATTTCACTTGCGGATCAGCCGAATAAGTACTATTTCATT  1766
                        ************************************************************

At2g28230|Col-0         ATCAGGACGCAGAGGATTGTCTTGGAAGCTGATTCTTCGATTCAGTTGATTATGG-AGAA  1847
At2g28230|Bg-2          ATCAGGACGCAGAGGATTGTCTTGGAAGCTGATTCTTCGATTCAGTTGATTATGG-AGAA  1842
At2g28230|Lm-2          ATCAGGACGCAGAGGATTGTCTTGGAAGCTGATTCTTCGATTCAGTTGATTATGG-AGAA  1826
At2g28230|Shahdara      ATCAGGACGCAGAGGATTGTCTTGGAAGCTGATTCTTCGATTCAGTTGATTATGG-AGAA  1826
At2g28230|TOU-I-17      ATCAGGACGCAGAGGATTGTCTTGGAAGCTGATTCTTCGATTCAGTTGATTATGG-AGAA  1847
At2g28230|Ag-0          ATCAGGACGCAGAGGATTGTCTTGGAAGCTGATTCTTCGATTCAGTTGATTATGG-AGAA  1847
At2g28230|Dra-2         ATCAGGACGCAGAGGATTGTCTTGGAAGCTGATTCTTCGATTCAGTTGATTATGG-AGAA  1859
At2g28230|UKSE06-414    ATCAGGACGCAGAGGATTGTCTTGGAAGCTGATTCTTCGATTCAGTTGATTATGG-AGAA  1825
At2g28230|Zdr-6         ATCAGGACGCAGAGGATTGTCTTGGAAGCTGATTCTTCGATTCAGTTGATTATGGGAGAA  1826
                        ******************************************************* ****

At2g28230|Col-0         GCTTCAGTCTTATAAATCTAAAGTGGCTCTTTACTTTGATGGGTTTCAGTATCAGCTTGG  1907
At2g28230|Bg-2          GCTCCAATCTTATAAATCGAAAGTGGCTCTTTACTTTGATGGGTTTCAGTATCAGCTTGG  1902
At2g28230|Lm-2          GCTCCAATCTTATAAATCGAAAGTGGCTCTTTACTTTGATGGGTTTCAGTATCAGCTTGG  1886
At2g28230|Shahdara      GCTTCAGTCTTATAAATCTAAAGTGGCTCTTTACTTTGATGGGTTTCAGTATCAGCTTGG  1886
At2g28230|TOU-I-17      GCTCCAATCTTATAAATCGAAAGTGGCTCTTTACTTTGATGGGTTTCAGTATCAGCTTGG  1907
At2g28230|Ag-0          GCTTCAGTCTTATAAATCTAAAGTGGCTCTTTACTTTGATGGGTTTCAGTATCAGCTTGG  1907
At2g28230|Dra-2         GCTTCAGTCTTATAAATCTAAAGTGGCTCTTTACTTTGATGGGTTTCAGTATCAGCTTGG  1919
At2g28230|UKSE06-414    GCTTCAGTCTTATAAATCTAAAGTGGCTCTTTACTTTGATGGGTTTCAGTATCAGCTTGG  1885
At2g28230|Zdr-6         GCTTCAGTCTTATAAATCTAAAGTGGCTCTTTACTTTGATGGGTTTCAGTATCAGCTTGG  1886
                        *** ** *********** *****************************************

At2g28230|Col-0         TGATTTTAGGTTGAGAGTTGGTAAAGTTGTTCCTACTCATTCTGAGAATGTTAGAGGCAT  1967
At2g28230|Bg-2          TGATTTTAGGTTGAGAGTTGGTAAAGTTGTTCCTACTCATTCTGAGAATGTTAGAGGCAT  1962
At2g28230|Lm-2          TGATTTTAGGTTGAGAGTTGGTAAAGTTGTTCCTACTCATTCTGAGAATGTTAGAGGCAT  1946
At2g28230|Shahdara      TGATTTTAGGTTGAGAGTTGGTAAAGTTGTTCCTACTCATTCTGAGAATGTTAGAGGCAT  1946
At2g28230|TOU-I-17      TGATTTTAGGTTGAGAGTTGGTAAAGTTGTTCCTACTCATTCTGAGAATGTTAGAGGCAT  1967
At2g28230|Ag-0          TGATTTTAGGTTGAGAGTTGGTAAAGTTGTTCCTACTCATTCTGAGAATGTTAGAGGCAT  1967
At2g28230|Dra-2         TGATTTTAGGTTGAGAGTTGGTAAAGTTGTTCCTACTCATTCTGAGAATGTTAGAGGCAT  1979
At2g28230|UKSE06-414    TGATTTTAGGTTGAGAGTTGGTAAAGTTGTTCCTACTCATTCTGAGAATGTTAGAGGCAT  1945
At2g28230|Zdr-6         TGATTTTAGGTTGAGAGTTGGTAAAGTTGTTCCTACTCATTCTGAGAATGTTAGAGGCAT  1946
                        ************************************************************

At2g28230|Col-0         TGTCATGGAGGTAAACAAAGTTTCCAGCTTTTTCTTGTTTAAGACAGATATGATGAACAC  2027
At2g28230|Bg-2          TGTCATGGAGGTAAACAAAGTTTCCAGCTTTTTCTTGTTTAAGACAGATATGATGAACAT  2022
At2g28230|Lm-2          TGTCATGGAGGTAAACAAAGTTTCCAGCTTTTTCTTGTTTAAGACAGATATGATGAACAT  2006
At2g28230|Shahdara      TGTCATGGAGGTAAACAAAGTTTCCAGCTTTTTCTTGTTATCGACATGCCTTTTTGGTTG  2006
At2g28230|TOU-I-17      TGTCATGGAGGTAAACAAAGTTTCCAGCTTTTTCTTGTTTAAGACAGATATGATGAACAT  2027
At2g28230|Ag-0          TGTCATGGAGGTAAACAAAGTTTCCAGCTTTTTCTTGTTTAAGACAGATATGATGAACAT  2027
At2g28230|Dra-2         TGTCATGGAGGTAAACAAAGTTTCCAGCTTTTTCTTGTTTAAGACAGATATGATGAACAT  2039
At2g28230|UKSE06-414    TGTCATGGAGGTAAACAAAGTTTCCAGCTTTTTCTTGTTTAAGACAGATATGATGAACAT  2005
At2g28230|Zdr-6         TGTCATGGAGGTAAACAAAGTTTCCAGCTTTTTCTTGTTTAAGACAGATATGATGAACAT  2006
                        ***************************************   ****    *  *

At2g28230|Col-0         TGAACAGTGGT-GATTAATTTTAAA---GATGAAAATCTTGTTTTTCTACAGATTTTTCT  2083
At2g28230|Bg-2          TGAACAGTGGT-GATTAATTTTAAA---GATGAAAATCTTGTTTTTCTACAGATTTTTCT  2078
At2g28230|Lm-2          TGAACAGTGGT-GATTAATTTTAAA---GATGAAAATCTTGTTTTTCTACAGATTTTTCT  2062
At2g28230|Shahdara      TGATTTAGTGATGATGATTCTTGATGCTGTTTAAAATGTTAGAGCTTGAGCAATTGA---  2063
At2g28230|TOU-I-17      TGAACAGGGGT-GATTAATTTTAAA---GATGAAAATCTTGTTTTTCTACAGATTTTTCT  2083
At2g28230|Ag-0          TGAACAGTGGT-GATTAATTTTAAA---GATGAAAATCTTGTTTTTCTACAGATTTTTCT  2083
At2g28230|Dra-2         TGAACAGTGGT-GATTAATTTTAAA---GATGAAAATCTTGTTTTTCTACAGATTTTTCT  2095
At2g28230|UKSE06-414    TGAACAGTGGT-GATTAATTTTAAA---GATGAAAATCTTGTTTTTCTACAGATTTTTCT  2061
At2g28230|Zdr-6         TGAACAGTGGT-GATTAATTTTAAA---GATGAAAATCTTGTTTTTCTACAGATTTTTCT  2062
                        ***      *  *** * * ** *    * * ***** **     *  *   ***

At2g28230|Col-0         TGTTAAAAGAGTTTCTTGTAATATCTTTTAGTGATGATCCTTGATGCTG-----------  2132
At2g28230|Bg-2          TGTTAAGAGAGTTTCTTGTAATATCTTTTAGTGATGATCCTTGATGCTG-----------  2127
At2g28230|Lm-2          TGTTAAGAGAGTTTCTTGTAATATCTTTTAGTGATGATCCTTGATGCTG-----------  2111
At2g28230|Shahdara      -----GACAGGTGCATAATAAT---GTTTGATGCGTTTAGTTGATAAAGTTCGTTTTCTA  2115
At2g28230|TOU-I-17      TGTTAAGAGAGTTTCTTGTAATATCTTTTAGTGATGATCCTTGATGCTG-----------  2132
At2g28230|Ag-0          TGTTAAGAGAGTTTCTTGTAATATCTTTTAGTGATGATCCTTGATGCTG-----------  2132
At2g28230|Dra-2         TGTTAAGAGAGTTTCTTGTAATATCTTTTAGTGATGATCCTTGATGCTG-----------  2144
At2g28230|UKSE06-414    TGTTAAGAGAGTTTCTTGTAATATCTTTTAGTGATGATCCTTGATGCTG-----------  2110
At2g28230|Zdr-6         TGTTAAGAGAGTTTCTTGTAATATCTTTTAGTGATGATCCTTGATGCTG-----------  2111
                                  **   *  ****    ***  **    *  *****   *

At2g28230|Col-0         ---TTTATGATGTTAAGACAGATGATGAAAATTGAACATTGTTGATTGTTGACTATGCAT  2189
At2g28230|Bg-2          ---TTTATGATGTTAAGACAGATGATGAAAATTGAACATTGTTGATTGTTGACTATGCAT  2184
At2g28230|Lm-2          ---TTTATGATGTTAAGACAGATGATGAAAATTGAACATTGTTGATTGTTGACTATGCAT  2168
At2g28230|Shahdara      GATTGTATATTGTTAGCAGAGTTTCTTGTAATCTCTTTTGGTGC--TATTTACAATGTTT  2173
At2g28230|TOU-I-17      ---TTTATGATGTTAAGACAGATGATGAAAATTGAACATTGTTGATTGTTGACTATGCAT  2189
```

```
At2g28230|Ag-0            ---TTTATGATGTTAAGACAGATGATGAAAATTGAACATTGTTGATTGTTGACTATGCAT  2189
At2g28230|Dra-2           ---TTTATGATGTTAAGACAGATGATGAAAATTGAACATTGTTGATTGTTGACTATGCAT  2201
At2g28230|UKSE06-414      ---TTTATGATGTNNNNNNNNNNNNNNNNNNNNNNNNNNNTGTTGATTGTTGACTATGCAT  2167
At2g28230|Zdr-6           ---TTTATGATGTTAAGACAGATGATGAAAATTGAACATTGTTGATTGTTGACTATGCAT  2168
                             * ***  ***                           **    * ** ** ***  *

At2g28230|Col-0           CTACA-AGTTTCTTTAGTCTTGTTTCGTGTAATCACAATGATGATGAAAAAATCCTTGTT  2248
At2g28230|Bg-2            CTACA-AGTTTCTTTAGTCTTGTTTCGTGTAATCACAATGATGATGAAAAAATCCTTGTT  2243
At2g28230|Lm-2            CTACA-AGTTTCTTTAGTCTTGTTTCGTGTAATCACAATGATGATGAAAAAATCCTTGTT  2227
At2g28230|Shahdara        AAGATAGATATGGTGAACATTGAACAGTGGTGATTCATTTTAAAGATGAAAATCCTG-TT  2232
At2g28230|TOU-I-17        CTACA-AGTTTCTTTAGTCTTGTTTCGTGTAATCACAATGATGATGAAAAAATCCTTGTT  2248
At2g28230|Ag-0            CTACA-AGTTTCTTTAGTCTTGTTTCGTGTAATCACAATGATGATGAAAAAATCCTTGTT  2248
At2g28230|Dra-2           CTACA-AGTTTCTTTAGTCTTGTTTCGTGTAATCACAATGATGATGAAAAAATCCTTGTT  2260
At2g28230|UKSE06-414      CTACA-AGTTTCTTTAGTCTTGTTTCGTGTAATCACAATGATGATGAAAAAATCCTTGTT  2226
At2g28230|Zdr-6           CTACA-AGTTTCTTTAGTCTTGTTTCGTGTAATCACAATGATGATGAAAAAATCCTTGTT  2227
                                  * *  * *   ***    ***      ** *    *    ********  **

At2g28230|Col-0           TTCTTAAATCCCTATAGTCTCAAT----GCTAATCTAATCACCTAAAGGGTATATGGAGC  2304
At2g28230|Bg-2            TTCTTAAATCCCTATAGTCTCAAT----GCTAATCTAATCACCTAAAGGGTATATGGAGC  2299
At2g28230|Lm-2            TTCTTAAATCCCTATAGTCTCAAT----GCTAATCTAATCACCTAAAGGGTATATGGAGC  2283
At2g28230|Shahdara        TTTCTACAGATTTTTCTTGTCAAAAGAGTTTCATGTAACCTCTTTTGGTGATGATCCCTC  2292
At2g28230|TOU-I-17        TTCTTAAATCCCTATAGTCTCAAT----GCTAATCTAATCACCTAAAGGGTATATGGAGC  2304
At2g28230|Ag-0            TTCTTAAATCCCTATAGTCTCAAT----GCTAATCTAATCACCTAAAGGGTATATGGAGC  2304
At2g28230|Dra-2           TTCTTAAATCCCTATAGTCTCAAT----GCTAATCTAATCACCTAAAGGGTATATGGAGC  2316
At2g28230|UKSE06-414      TTCTTAAATCCCTATAGTCTCAAT----GCTAATCTAATCACCTAAAGGGTATATGGAGC  2282
At2g28230|Zdr-6           TTCTTAAATCCCTATAGTCTCAAT----GCTAATCTAATCACCTAAAGGGTATATGGAGC  2283
                          **  ** *    * *  * ****       * ** *** * * *   * *   **    *

At2g28230|Col-0           TGTTCCCTTTTGGTGATTGTCTTGTTTCTTGTAATCTCAATTTTGAGTTTGTGTGTTGTT  2364
At2g28230|Bg-2            TGTTCCCTTTTGGTGATTGTCTTGTTTCTTGTAATCTCAATTTTGAGTTTGTGTGTTGTT  2359
At2g28230|Lm-2            TGTTCCCTTTTGGTGATTGTCTTGTTTCTTGTAATCTCAATTTTGAGTTTGTGTGTTGTT  2343
At2g28230|Shahdara        GATGCTGTTTATG-ATGTTAAGACAGATGATGAAAATTGAACTAAAGGATGTGTGTGTAT  2351
At2g28230|TOU-I-17        TGTTCCCTTTTGGTGATTGTCTTGTTTCTTGTAATCTCAATTTTGAGTTTGTGTGTTGTT  2364
At2g28230|Ag-0            TGTTCCCTTTTGGTGATTGTCTTGTTTCTTGTAATCTCAATTTTGAGTTTGTGTGTTGTT  2364
At2g28230|Dra-2           TGTTCCCTTTTGGTGATTGTCTTGTTTCTTGTAATCTCAATTTTGAGTTTGTGTGTTGTT  2376
At2g28230|UKSE06-414      TGTTCCCTTTTGGTGATTGTCTTGTTTCTTGTAATCTCAATTTTGAGTTTGTGTGTTGTT  2342
At2g28230|Zdr-6           TGTTCCCTTTTGGTGATTGTCTTGTTTCTTGTAATCTCAATTTTGAGTTTGTGTGTTGTT  2343
                            * *  ***  *    *              **  *  *  *  **  *******   *

At2g28230|Col-0           ACTTGCAGGTGGAGTATCTTCCTATATCATCAATGGAAAAGGCACAAAAGGTGATGGAGG  2424
At2g28230|Bg-2            ACTTGCAGGTGGAGTATCTTCCTATATCATCAATGGAAAAGGCACAAAAGGTGATGGAGG  2419
At2g28230|Lm-2            ACTTGCAGGTGGAGTATCTTCCTATATCATCAATGGAAAAGGCACAAAAGGTGATGGAGG  2403
At2g28230|Shahdara        CCTTGCAGGTGGAGTATCTTCCTATATCATCAATGGAAAAGGCACAAAAAGTGATGGAGG  2411
At2g28230|TOU-I-17        ACTTGCAGGTGGAGTATCTTCCTATATCATCAATGGAAAAGGCACAAAAGGTGATGGAGG  2424
At2g28230|Ag-0            ACTTGCAGGTGGAGTATCTTCCTATATCATCAATGGAAAAGGCACAAAAGGTGATGGAGG  2424
At2g28230|Dra-2           ACTTGCAGGTGGAGTATCTTCCTATATCATCAATGGAAAAGGCACAAAAGGTGATGGAGG  2436
At2g28230|UKSE06-414      ACTTGCAGGTGGAGTATCTTCCTATATCATCAATGGAAAAGGCACAAAAGGTGATGGAGG  2402
At2g28230|Zdr-6           ACTTGCAGGTGGAGTATCTTCCTATATCATCAATGGAAAAGGCACAAAAGGTGATGGAGG  2403
                           ************************************************ **********

At2g28230|Col-0           AGTTCTTGGAGATATGGAATGAAGCTCTGGCTAAAAGGTCGTTGCCGGGTAAGTTTGTGA  2484
At2g28230|Bg-2            AGTTCTTGGAGATATGGAATGAAGCTCTGGCTAAAAGGTCGTTGCCGGGTAAGTTTGTGA  2479
At2g28230|Lm-2            AGTTCTTGGAGATATGGAATGAAGCTCTGGCTAAAAGGTCGTTGCCGGGTAAGTTTGTGA  2463
At2g28230|Shahdara        AGTTCTTGGAGATATGGAATGAAGCTCTGGCTAAAAGGTCGTTGCCGGGTAAGTTTGTGA  2471
At2g28230|TOU-I-17        AGTTCTTGGAGATATGGAATGAAGCTCTGGCTAAAAGGTCGTTGCCGGGTAAGTTTGTGA  2484
At2g28230|Ag-0            AGTTCTTGGAGATATGGAATGAAGCTCTGGCTAAAAGGTCGTTGCCGGGTAAGTTTGTGA  2484
At2g28230|Dra-2           AGTTCTTGGAGATATGGAATGAAGCTCTGGCTAAAAGGTCGTTGCCGGGTAAGTTTGTGA  2496
At2g28230|UKSE06-414      AGTTCTTGGAGATATGGAATGAAGCTCTGGCTAAAAGGTCGTTGCCGGGTAAGTTTGTGA  2462
At2g28230|Zdr-6           AGTTCTTGGAGATATGGAATGAAGCTCTGGCTAAAAGGTCGTTGCCGGGTAAGTTTGTGA  2463
                          ************************************************************

At2g28230|Col-0           ACATAGATCTCAACTTTGGGGAGTTTGGACTTGGAGACATCTACACTCCACAACACACAG  2544
At2g28230|Bg-2            ACATAGATCTCAACTTTGGGGAGTTTGGACTTGGAGACATCTACACTCCACAACACACAG  2539
At2g28230|Lm-2            ACATAGATCTCAACTTTGGGGAGTTTGGACTTGGAGACATCTACACTCCACAACACACAG  2523
At2g28230|Shahdara        ACATAGATCTCAACTTTGGGGAGTTTGGACTTGGAGACATCTACACTCCACAGCACACAG  2531
At2g28230|TOU-I-17        ACATAGATCTCAACTTTGGGGAGTTTGGACTTGGAGACATCTACACTCCACAACACACAG  2544
At2g28230|Ag-0            ACATAGATCTCAACTTTGGGGAGTTTGGACTTGGAGACATCTACACTCCACAACACACAG  2544
At2g28230|Dra-2           ACATAGATCTCAACTTTGGGGAGTTTGGACTTGGAGACATCTACACTCCACAACACACAG  2556
At2g28230|UKSE06-414      ACATAGATCTCAACTTTGGGGAGTTTGGACTTGGAGACATCTACACTCCACAACACACAG  2522
At2g28230|Zdr-6           ACATAGATCTCAACTTTGGGGAGTTTGGACTTGGAGACATCTACACTCCACAACACACAG  2523
                          **************************************************** *******

At2g28230|Col-0           CTGTTCGTTACGCTCTCGTGATGGCTCACATGATTGCTACCGTTCAAGCTGTGAGAGGCT  2604
At2g28230|Bg-2            CTGTTCGTTACGCTCTCGTGATGGCTCACATGATTGCTACCGTTCAAGCTGTGAGAGGCT  2599
At2g28230|Lm-2            CTGTTCGTTACGCTCTCGTGATGGCTCACATGATTGCTACCGTTCAAGCTGTGAGAGGCT  2583
At2g28230|Shahdara        CTGTTCGTTACGCTCTCGTTATGGCTCACATGAATGCTACCGTTCAAGCTGTGAGAGGCT  2591
At2g28230|TOU-I-17        CTGTTCGTTACGCTCTCGTGATGGCTCACATGATTGCTACCGTTCAAGCTGTGAGAGGCT  2604
At2g28230|Ag-0            CTGTTCGTTACGCTCTCGTGATGGCTCACATGATTGCTACCGTTCAAGCTGTGAGAGGCT  2604
At2g28230|Dra-2           CTGTTCGTTACGCTCTCGTGATGGCTCACATGATTGCTACCGTTCAAGCTGTGAGAGGCT  2616
At2g28230|UKSE06-414      CTGTTCGTTACGCTCTCGTGATGGCTCACATGATTGCTACCGTTCAAGCTGTGAGAGGCT  2582
At2g28230|Zdr-6           CTGTTCGTTACGCTCTCGTGATGGCTCACATGATTGCTACCGTTCAAGCTGTGAGAGGCT  2583
```

```
                                    ******************* ************* **************************

At2g28230|Col-0                     AAACCAAAACATAACTTAGTATTTCTAGGTAAAATTATGTGCTTTGCAATATTAGTGATT  2664
At2g28230|Bg-2                      AAACCAAAACATAACTTAGTATTTCTAGGTAAAATTATGTGCTTTGCAATATTAGTGATT  2659
At2g28230|Lm-2                      AAACCAAAACATAACTTAGTATTTCTAGGTAAAATTATGTGCTTTGCAATATTAGTGATT  2643
At2g28230|Shahdara                  AAACCAAAACTCAGCTTTGTATTTAGGTATA--ATTCTGTGTTTTGCATTATTAGTGATT  2649
At2g28230|TOU-I-17                  AAACCAAAACATAACTTAGTATTTCTAGGTAAAATTATGTGCTTTGCAATATTAGTGATT  2664
At2g28230|Ag-0                      AAACCAAAACATAACTTAGTATTTCTAGGTAAAATTATGTGCTTTGCAATATTAGTGATT  2664
At2g28230|Dra-2                     AAACCAAAACATAACTTAGTATTTCTAGGTAAAATTATGTGCTTTGCAATATTAGTGATT  2676
At2g28230|UKSE06-414                AAACCAAAACATAACTTAGTATTTCTAGGTAAAATTATGTGCTTTGCAATATTAGTGATT  2642
At2g28230|Zdr-6                     AAACCAAAACATAACTTAGTATTTCTAGGTAAAATTATGTGCTTTGCAATATTAGTGATT  2643
                                    **********  * *** ******     **  *** **** ****** ***********

At2g28230|Col-0                     TCTTT----CTTTGGTCATGTTTATCTATAAGAAGCATTGAGAAAAAATGAACTTATAAA  2720
At2g28230|Bg-2                      TCTTT----CTTTGGTCATGTTTATCTATAAGAAGCATTGAGAAAAAATGAACTTATAAA  2715
At2g28230|Lm-2                      TCTTT----CTTTGGTCATGTTTATCTATAAGAAGCATTGAGAAAAAATGAACTTATAAA  2699
At2g28230|Shahdara                  TCGTTAGCCATGTTTATCAGTTTATCTATAAGAAGCATTGAGAAAATATGAACTTTTAAA  2709
At2g28230|TOU-I-17                  TCTTT----CTTTGGTCATGTTTATCTATAAGAAGCATTGAGAAAAAATGAACTTATAAA  2720
At2g28230|Ag-0                      TCTTT----CTTTGGTCATGTTTATCTATAAGAAGCATTGAGAAAAAATGAACTTATAAA  2720
At2g28230|Dra-2                     TCTTT----CTTTGGTCATGTTTATCTATAAGAAGCATTGAGAAAAAATGAACTTATAAA  2732
At2g28230|UKSE06-414                TCTTT----CTTTGGTCATGTTTATCTATAAGAAGCATTGAGAAAAAATGAACTTATAAA  2698
At2g28230|Zdr-6                     TCTTT----CTTTGGTCATGTTTATCTATAAGAAGCATTGAGAAAAAATGAACTTATAAA  2699
                                    ** **     * *      *************************** ******** ****

At2g28230|Col-0                     AGACTTTGATAGAACAAGTTGCAAACTCCAGAAAAAAACTTGAGATTATAGCAGATTGTGA  2780
At2g28230|Bg-2                      AGACTTTGATAGAACAAGTTGCAAACTCCAGAAAAAAACTTGAGATTATAGCAGATTGTGA  2775
At2g28230|Lm-2                      AGACTTTGATAGAACAAGTTGCAAACTCCAGAAAAAAACTTGAGATTGTAGCAGATTGTGA  2759
At2g28230|Shahdara                  AGACTTTGATAGAACAAGTTGCAAACTCCAGAAAAAAACTTGAGATTGTAGCAGATTGTGA  2769
At2g28230|TOU-I-17                  AGACTTTGATAGAACAAGTTGCAAACTCCAGAAAAAAACTTGAGATTATAGCAGATTGTGA  2780
At2g28230|Ag-0                      AGACTTTGATAGAACAAGTTGCAAACTCCAGAAAAAAACTTGAGATTATAGCAGATTGTGA  2780
At2g28230|Dra-2                     AGACTTTGATAGAACAAGTTGCAAACTCCAGAAAAAAACTTGAGATTATAGCAGATTGTGA  2792
At2g28230|UKSE06-414                AGACTTTGATAGAACAAGTTGCAAANNNNNNNNNNNNNNNNNNNNNNNAGCAGATTGTGA  2758
At2g28230|Zdr-6                     AGACTTTGATAGAACAAGTTGCAAACTCCAGAAAAAAACTTGAGATTATAGCAGATTGTGA  2759
                                    *************************                       ************

At2g28230|Col-0                     TTT--GAATTTCGATTAATTGTGTAACTCATTCGCATATTTTTTCAT-TTAAACATGTCT  2837
At2g28230|Bg-2                      TTT--GAATTTCGATTAATTGTGTAACTCATTCGCATATTTTTTCAT-TTAAACATGTCT  2832
At2g28230|Lm-2                      TTT--GAATTTCGATTAATTGTGTAACTCATTCGCATATTTTTTCAT-TTAAACATGTCT  2816
At2g28230|Shahdara                  TTT--GAATTTCGATTAATTGTGTAACTCATTCGCATATTCTTTCATTTAAAACATGTCT  2827
At2g28230|TOU-I-17                  TTT--GAATTTCGATTAATTGTGTAACTCATTCGCATATTTTTTCAT-TTAAACATGTCT  2837
At2g28230|Ag-0                      TTT--GAATTTCGATTAATTGTGTAACTCATTCGCATATTTTTTCAT-TTAAACATGTCT  2837
At2g28230|Dra-2                     TTTGGGAATTTCGATTAATTGTGTAACTCATTCGCATATTTTTTCAT-TTAAACATGTCT  2851
At2g28230|UKSE06-414                T--TTGAATTTCGATTAATTGTGTAACTCATTCGCATATTTTTTCAT-TTAAACATGTCT  2815
At2g28230|Zdr-6                     TTT--GAATTTCGATTAATTGTGTAACTCATTCGCATATTTTTTCAT-TTAAACATGTCT  2816
                                    *    *********************************** ****** * **********

At2g28230|Col-0                     ATGTCATATGCTTTTAGTGACAGTACAAATAAATCGAAACAAAACTGAAGTTAAAACCAT  2897
At2g28230|Bg-2                      ATGTCATATGCTTTTAGTGACAGTACAAATAAATCGAAACAAAACTGAAGTTAAAACCAT  2892
At2g28230|Lm-2                      ATGTCATATGCTTTTAGTGACAGTACAAATAAATCGAAACAAAACTGAAGTTAAAACCAT  2876
At2g28230|Shahdara                  ATGTCATATGCTAATAGTGACAGTACAAATAAATCGAAACAAAACTTAAGTTAAAACCAT  2887
At2g28230|TOU-I-17                  ATGTCATATGCTTTTAGTGACAGTACAAATAAATCGAAACAAAACTGAAGTTAAAACCAT  2897
At2g28230|Ag-0                      ATGTCATATGCTTTTAGTGACAGTACAAATAAATCGAAACAAAACTGAAGTTAAAACCAT  2897
At2g28230|Dra-2                     ATGTCATATGCTTTTAGTGACAGTACAAATAAATCGAAACAAAACTGAAGTTAAAACCAT  2911
At2g28230|UKSE06-414                ATGTCATATGCTTTTAGTGACAGTACAAATAAATCGAAACAAAACTGAAGTTAAAACCAT  2875
At2g28230|Zdr-6                     ATGTCATATGCTTTTAGTGACAGTACAAATAAATCGAAACAAAACTGAAGTTAAAACCAT  2876
                                    ************  ******************************** *************

At2g28230|Col-0                     GAAAATGTAGAAAAGAGAATCTCTTGGTTTGTTTCTTCAACTCCACAAAGCAGAACAATT  2957
At2g28230|Bg-2                      GAAAATGTAGAAAAGAGAATCTCTTGGTTTGTTTCTTCAACTCCACAAAGCAGAACAATT  2952
At2g28230|Lm-2                      GAAAATGTAGAAAAGAGAATCTCTTGGTTTGTTTCTTCAACTCCACAAAGCAGAACAATT  2936
At2g28230|Shahdara                  GAAAATGTAGAAAAGAGATTCTCTTGGTTTGTTTCTTCAACTCCACAATGCAGAACAATT  2947
At2g28230|TOU-I-17                  GAAAATGTAGAAAAGAGAATCTCTTGGTTTGTTTCTTCAACTCCACAAAGCAGAACAATT  2957
At2g28230|Ag-0                      GAAAATGTAGAAAAGAGAATCTCTTGGTTTGTTTCTTCAACTCCACAAAGCAGAACAATT  2957
At2g28230|Dra-2                     GAAAATGTAGAAAAGAGAATCTCTTGGTTTGTTTCTTCAACTCCACAAAGCAGAACAATT  2971
At2g28230|UKSE06-414                GAAAATGTAGAAAAGAGAATCTCTTGGTTTGTTTCTTCAACTCCACAAAGCAGAACAATT  2935
At2g28230|Zdr-6                     GAAAATGTAGAAAAGAGAATCTCTTGGTTTGTTTCTTCAACTCCACAAAGCAGAACAATT  2936
                                    ****************** ***************************** ***********

At2g28230|Col-0                     AGTGGGACTGAAAGAAATCACATTTGAAGAAGGATCATAACCCACCAAAAAATTGTTCTG  3017
At2g28230|Bg-2                      AGTGGGACTGAAAGAAATCACATTTGAAGAAGGATCATAACCCACCAAAAAATTGTTCTG  3012
At2g28230|Lm-2                      AGTGGGACTGAAAGAAATCACATTTGAAGAAGGATCATAACCCACCAAAAAATTGTTCTG  2996
At2g28230|Shahdara                  AGTGGGACTGAAAGAAATCACATTTGAAGAAGGGTCATAACCCACCAAAAAATTGTTCTG  3007
At2g28230|TOU-I-17                  AGTGGGACTGAAAGAAATCACATTTGAAGAAGGATCATAACCCACCAAAAAATTGTTCTG  3017
At2g28230|Ag-0                      AGTGGGACTGAAAGAAATCACATTTGAAGAAGGATCATAACCCACCAAAAAATTGTTCTG  3017
At2g28230|Dra-2                     AGTGGGACTGAAAGAAATCACATTTGAAGAAGGATCATAACCCACCAAAAAATTGTTCTG  3031
At2g28230|UKSE06-414                AGTGGGACTGAAAGAAATCACATTTGAAGAAGGATCATAACCCACCAAAAAATTGTTCTG  2995
At2g28230|Zdr-6                     AGTGGGACTGAAAGAAATCACATTTGAAGAAGGATCATAACCCACCAAAAAATTGTTCTG  2996
                                    ********************************* **************************

At2g28230|Col-0                     CGCTCTGTTCCCAAAGACAGCAGGCATTGACGGATCATTACACCCAATAGCCAAACAAAA  3077
At2g28230|Bg-2                      CGCTCTGTTCCCAAAGACAGCAGGCATTGACGGATCATTACACCCAATAGCCAAACAAAA  3072
```

```
At2g28230|Lm-2           CGCTCTGTTCCCAAAGACAGCAGGCATTGACGGATCATTACACCCAATAGCCAAACAAAA  3056
At2g28230|Shahdara       CGCTCTGTTCCCAAAGACAGCAGGCATTGTCGGATCATTACACCCAATAGCCAAACAAAA  3067
At2g28230|TOU-I-17       CGCTCTGTTCCCAAAGACAGCAGGCATTGACGGATCATTACACCCAATAGCCAAACAAAA  3077
At2g28230|Ag-0           CGCTCTGTTCCCAAAGACAGCAGGCATTGACGGATCATTACACCCAATAGCCAAACAAAA  3077
At2g28230|Dra-2          CGCTCTGTTCCCAAAGACAGCAGGCATTGACGGATCATTACACCCAATAGCCAAACAAAA  3091
At2g28230|UKSE06-414     CGCTCTGTTCCCAAAGACAGCAGGCATTGACGGATCATTACACCCAATAGCCAAACAAAA  3055
At2g28230|Zdr-6          CGCTCTGTTCCCAAAGACAGCAGGCATTGACGGATCATTACACCCAATAGCCAAACAAAA  3056
                         ***************************** ******************************

At2g28230|Col-0          GATTCCTCCGGTAATCGTTTCGAGATACATATTATACTTATCCAAGACAAGATCCGCACC  3137
At2g28230|Bg-2           GATTCCTCCGGTAATCGTTTCGAGATACATATTATACTTATCCAAGACAAGATCCGCACC  3132
At2g28230|Lm-2           GATTCCTCCGGTAATCGTTTCGAGATACATATTATACTTATCCAAGACAAGATCCGCACC  3116
At2g28230|Shahdara       GATTCCTCCCGTAATCGTTTCGAGATACATATTATACTTATCCAAGACAAGATCCGCACC  3127
At2g28230|TOU-I-17       GATTCCTCCGGTAATCGTTTCGAGATACATATTATACTTATCCAAGACAAGATCCGCACC  3137
At2g28230|Ag-0           GATTCCTCCGGTAATCGTTTCGAGATACATATTATACTTATCCAAGACAAGATCCGCACC  3137
At2g28230|Dra-2          GATTCCTCCGGTAATCGTTTCGAGATACATATTATACTTATCCAAGACAAGATCCGCACC  3151
At2g28230|UKSE06-414     GATTCCTCCGGTAATCGTTTCGAGATACATATTATACTTATCCAAGACAAGATCCGCACC  3115
At2g28230|Zdr-6          GATTCCTCCGGTAATCGTTTCGAGATACATATTATACTTATCCAAGACAAGATCCGCACC  3116
                         ********* **************************************************

At2g28230|Col-0          GCCAGAAAAATGCATTGTGATCACGGGAAAGATATCTATGGTGTCCGAGTAGTAGCAAAG  3197
At2g28230|Bg-2           GCCAGAAAAATGCATTGTGATCATGGGAAAGATATCTATGGTGTCCGAGTAGTAGCAAAG  3192
At2g28230|Lm-2           GCCAGAAAAATGCATTGTGATCATGGGAAAGATATCTATGGTGTCCGAGTAGTAGCAAAG  3176
At2g28230|Shahdara       GCCAGAAAAATGCATTGTGATCACCGGAAAGATATCTATGGTGTCCGAGTAGTAGCAAAG  3187
At2g28230|TOU-I-17       GCCAGAAAAATGCATTGTGATCATGGGAAAGATATCTATGGTGTCCGAGTAGTAGCAAAG  3197
At2g28230|Ag-0           GCCAGAAAAATGCATTGTGATCACGGGAAAGATATCTATGGTGTCCGAGTAGTAGCAAAG  3197
At2g28230|Dra-2          GCCAGAAAAATGCATTGTGATCACGGGAAAGATATCTATGGTGTCCGAGTAGTAGCAAAG  3211
At2g28230|UKSE06-414     GCCAGAAAAATGCATTGTGATCACGGGAAAGATATCTATGGTGTCCGAGTAGTAGCAAAG  3175
At2g28230|Zdr-6          GCCAGAAAAATGCATTGTGATCACGGGAAAGATATCTATGGTGTCCGAGTAGTAGCAAAG  3176
                         ***********************  ***********************************

At2g28230|Col-0          CAAGTTGTCACTGCCCATGTCAGGTACTTTAACTGCTGTCACAACCTGTTCCACTGCCTC  3257
At2g28230|Bg-2           CAAGTTGTCACTGCCCATGTCAGGTACTTTAACTGCTGTCACAACCTGTTCCACTGCCTC  3252
At2g28230|Lm-2           CAAGTTGTCACTGCCCATGTCAGGTACTTTAACTGCTGTCACAACCTGTTCCACTGCCTC  3236
At2g28230|Shahdara       CAAGTTGTCACTGCCCATGTCAGGTACTTGAACTGCTGTCACAACCTGTTCCACTGCCTC  3247
At2g28230|TOU-I-17       CAAGTTGTCACTGCCCATGTCAGGTACTTTAACTGCTGTCACAACCTGTTCCACTGCCTC  3257
At2g28230|Ag-0           CAAGTTGTCACTGCCCATGTCAGGTACTTTAACTGCTGTCACAACCTGTTCCACTGCCTC  3257
At2g28230|Dra-2          CAAGTTGTCACTGCCCATGTCAGGTACTTTAACTGCTGTCACAACCTGTTCCACTGCCTC  3271
At2g28230|UKSE06-414     CAAGTTGTCACTGCCCATGTCAGGTACTTTAACTGCTGTCACAACCTGTTCCACTGCCTC  3235
At2g28230|Zdr-6          CAAGTTGTCACTGCCCATGTCAGGTACTTTAACTGCTGTCACAACCTGTTCCACTGCCTC  3236
                         ***************************** ******************************

At2g28230|Col-0          CCTCACTAGGTTGCAGTAGCTCATTGGAAAGTACGTGAGAGTGGTTCCTGAGTCTATAAA  3317
At2g28230|Bg-2           CCTCACTAGGTTGCAGTAGCTCATTGGAAAGTACGTGAGAGTGGTTCCTGAG--------  3304
At2g28230|Lm-2           CCTCACTAGGTTGCAGTAGCTCATTGGAAAGTACGTGAGAGTGGTTCCTGAG--------  3288
At2g28230|Shahdara       CCTCACTAGGTTGCAGTAGCTCATTGGAAAGTACGTGAGAGTGGTTCCTGAG--------  3299
At2g28230|TOU-I-17       CCTCACTAGGTTGCAGTAGCTCATTGGAAAGTACGTGAGAGTGGTTCCTGAG--------  3309
At2g28230|Ag-0           CCTCACTAGGTTGCAGTAGCTCATTGGAAAGTACGTGAGAGTGGTTCCTGAG--------  3309
At2g28230|Dra-2          CCTCACTAGGTTGCANNNNNNNNNNNNNNNNNNNNNNNNNNNNNNNNNNNNN--------  3323
At2g28230|UKSE06-414     CCTCACTAGGTTGCAGTAGCTCATTGGAAAGTACGTGAGAGTGGTTCCTGAG--------  3287
At2g28230|Zdr-6          CCTCACTAGGTTGCAGTAGCTCATTGGAAAGTACGTGAGAGTGGTTCCTGAG--------  3288
                         ***************

At2g28230|Col-0          AATGTTCCCGTCTTCGGCATGAAACGGTGTCCCCAATGTCGCAATCAGGTTGTCCTCAAC  3377
At2g28230|Bg-2           ------------------------------------------------------------  3304
At2g28230|Lm-2           ------------------------------------------------------------  3288
At2g28230|Shahdara       ------------------------------------------------------------  3299
At2g28230|TOU-I-17       ------------------------------------------------------------  3309
At2g28230|Ag-0           ------------------------------------------------------------  3309
At2g28230|Dra-2          ------------------------------------------------------------  3323
At2g28230|UKSE06-414     ------------------------------------------------------------  3287
At2g28230|Zdr-6          ------------------------------------------------------------  3288

At2g28230|Col-0          GCTGACCGCGTCTAGGTTTAGATAATAGAAAGGATTATCTTTCTTGATAAACATATCGGC  3437
At2g28230|Bg-2           ------------------------------------------------------------  3304
At2g28230|Lm-2           ------------------------------------------------------------  3288
At2g28230|Shahdara       ------------------------------------------------------------  3299
At2g28230|TOU-I-17       ------------------------------------------------------------  3309
At2g28230|Ag-0           ------------------------------------------------------------  3309
At2g28230|Dra-2          ------------------------------------------------------------  3323
At2g28230|UKSE06-414     ------------------------------------------------------------  3287
At2g28230|Zdr-6          ------------------------------------------------------------  3288

At2g28230|Col-0          TGCTACCGTTCCATCTCCTGCCACAATGGCATTTGTTCCAAAATTGATCTTACTAGTTCC  3497
At2g28230|Bg-2           ------------------------------------------------------------  3304
At2g28230|Lm-2           ------------------------------------------------------------  3288
At2g28230|Shahdara       ------------------------------------------------------------  3299
At2g28230|TOU-I-17       ------------------------------------------------------------  3309
At2g28230|Ag-0           ------------------------------------------------------------  3309
```

```
At2g28230|Dra-2         ------------------------------------------------------------ 3323
At2g28230|UKSE06-414    ------------------------------------------------------------ 3287
At2g28230|Zdr-6         ------------------------------------------------------------ 3288

At2g28230|Col-0         TTGACCAGAAAAGCAGTAAGAGATAAGACCTGGGTAGGGTAAGTCCATCTGAGATATAAG 3557
At2g28230|Bg-2          ------------------------------------------------------------ 3304
At2g28230|Lm-2          ------------------------------------------------------------ 3288
At2g28230|Shahdara      ------------------------------------------------------------ 3299
At2g28230|TOU-I-17      ------------------------------------------------------------ 3309
At2g28230|Ag-0          ------------------------------------------------------------ 3309
At2g28230|Dra-2         ------------------------------------------------------------ 3323
At2g28230|UKSE06-414    ------------------------------------------------------------ 3287
At2g28230|Zdr-6         ------------------------------------------------------------ 3288

At2g28230|Col-0         CGACAAAGGTCCCATATTTAGACCAACAATGCCGGAAGAACTCGATGCAAACCCTGAGTA 3617
At2g28230|Bg-2          ------------------------------------------------------------ 3304
At2g28230|Lm-2          ------------------------------------------------------------ 3288
At2g28230|Shahdara      ------------------------------------------------------------ 3299
At2g28230|TOU-I-17      ------------------------------------------------------------ 3309
At2g28230|Ag-0          ------------------------------------------------------------ 3309
At2g28230|Dra-2         ------------------------------------------------------------ 3323
At2g28230|UKSE06-414    ------------------------------------------------------------ 3287
At2g28230|Zdr-6         ------------------------------------------------------------ 3288

At2g28230|Col-0         CTGGAGGTTTGTGTTGTCGAGGCCACAACCAATTTTTGTTTCAGCCATCACAAAGGGCTC 3677
At2g28230|Bg-2          ------------------------------------------------------------ 3304
At2g28230|Lm-2          ------------------------------------------------------------ 3288
At2g28230|Shahdara      ------------------------------------------------------------ 3299
At2g28230|TOU-I-17      ------------------------------------------------------------ 3309
At2g28230|Ag-0          ------------------------------------------------------------ 3309
At2g28230|Dra-2         ------------------------------------------------------------ 3323
At2g28230|UKSE06-414    ------------------------------------------------------------ 3287
At2g28230|Zdr-6         ------------------------------------------------------------ 3288

At2g28230|Col-0         TCCTGAAGTGGAAGGTATCGTGACCGTCTCGGTTGCCAAGATTCCCTTGGAATAGGTTTT 3737
At2g28230|Bg-2          ------------------------------------------------------------ 3304
At2g28230|Lm-2          ------------------------------------------------------------ 3288
At2g28230|Shahdara      ------------------------------------------------------------ 3299
At2g28230|TOU-I-17      ------------------------------------------------------------ 3309
At2g28230|Ag-0          ------------------------------------------------------------ 3309
At2g28230|Dra-2         ------------------------------------------------------------ 3323
At2g28230|UKSE06-414    ------------------------------------------------------------ 3287
At2g28230|Zdr-6         ------------------------------------------------------------ 3288

At2g28230|Col-0         GTCCGCGTAGATAATCTCGTAATGACAAGAGTTTCCATTGCATCTTTGTTCTCTGAAGGT 3797
At2g28230|Bg-2          ------------------------------------------------------------ 3304
At2g28230|Lm-2          ------------------------------------------------------------ 3288
At2g28230|Shahdara      ------------------------------------------------------------ 3299
At2g28230|TOU-I-17      ------------------------------------------------------------ 3309
At2g28230|Ag-0          ------------------------------------------------------------ 3309
At2g28230|Dra-2         ------------------------------------------------------------ 3323
At2g28230|UKSE06-414    ------------------------------------------------------------ 3287
At2g28230|Zdr-6         ------------------------------------------------------------ 3288

At2g28230|Col-0         TGAAGATTTCGAAGGGTCGAATATAGGAGCGAATTGG 3834
At2g28230|Bg-2          ------------------------------------- 3304
At2g28230|Lm-2          ------------------------------------- 3288
At2g28230|Shahdara      ------------------------------------- 3299
At2g28230|TOU-I-17      ------------------------------------- 3309
At2g28230|Ag-0          ------------------------------------- 3309
At2g28230|Dra-2         ------------------------------------- 3323
At2g28230|UKSE06-414    ------------------------------------- 3287
At2g28230|Zdr-6         ------------------------------------- 3288
```
