# Supplementary material for: Natural expression variation for the Arabidopsis MED20a mediator complex subunit influences quantitative resistance to Sclerotinia sclerotiorum
Source: Front Plant Sci. 2025 Nov 17;16:1706963. doi: 10.3389/fpls.2025.1706963 (PMC12667439; doi:10.3389/fpls.2025.1706963)
Supplement: Supplementary Table 2 — Primers for Sanger sequencing of MED20a from nine A. thaliana ecotypes. [file Table2.docx]

Table S2. Primers for Sanger sequencing of *MED20a* from six *A. thaliana* ecotypes.

| **Primer** | **Sequence** |
| --- | --- |
| Med20a Fwd 1 | TCCCTTGTTCTCTGCTCAGC |
| Med20a Rev 1 | TCGTTTTCCAGACCATGCCT |
| Med20a Fwd 2 | GAGGCATGGTCTGGAAAACG |
| Med20a Rev 2 | TGGTTCTTCACAATCTAGCATATATGA |
| Med20a Fwd 3 | TCATATATGCTAGATTGTGAAGAACCA |
| Med20a Rev 3 | TGGCTGCTTACTGTTGATCC |
| Med20a Fwd 4 | GGATCAACAGTAAGCAGCCA |
| Med20a Rev 4 | ACATCATAAACAGCATCAAGGATCA |
| Med20a Fwd 5 | TGATCCTTGATGCTGTTTATGATGT |
| Med20a Rev 5 | TCGAAATTCAAATCACAATCTGCT |
| Med20a Fwd 6 | AGCAGATTGTGATTTGAATTTCGA |
| Med20a Rev 6 | CTCAGGAACCACTCTCACGT |
